# Bayesian model selection for spatial capture–recapture models

**DOI:** 10.1002/ece3.5551

**Published:** 2019-09-30

**Authors:** Soumen Dey, Mohan Delampady, Arjun M. Gopalaswamy

**Affiliations:** ^1^ Statistics and Mathematics Unit Indian Statistical Institute Bangalore India

**Keywords:** Bayes factors, Bayesian inference, DIC, hierarchical models, posterior predictive loss, WAIC

## Abstract

A vast amount of ecological knowledge generated over the past two decades has hinged upon the ability of model selection methods to discriminate among various ecological hypotheses. The last decade has seen the rise of Bayesian hierarchical models in ecology. Consequently, commonly used tools, such as the AIC, become largely inapplicable and there appears to be no consensus about *a* particular model selection tool that can be universally applied. We focus on a specific class of competing Bayesian spatial capture–recapture (SCR) models and apply and evaluate some of the recommended Bayesian model selection tools: (1) Bayes Factor—using (a) Gelfand‐Dey and (b) harmonic mean methods, (2) Deviance Information Criterion (DIC), (3) Watanabe‐Akaike's Information Criterion (WAIC) and (4) posterior predictive loss criterion. In all, we evaluate 25 variants of model selection tools in our study. We evaluate these model selection tools from the standpoint of selecting the “true” model and parameter estimation. In all, we generate 120 simulated data sets using the true model and assess the frequency with which the true model is selected and how well the tool estimates *N* (population size), a parameter of much importance to ecologists. We find that when information content is low in the data, no particular model selection tool can be recommended to help realize, simultaneously, both the goals of model selection and parameter estimation. But, in general (when we consider both the objectives together), we recommend the use of our application of the Bayes Factor (Gelfand‐Dey with MAP approximation) for Bayesian SCR models. Our study highlights the point that although new model selection tools are emerging (e.g., WAIC) in the applied statistics literature, those tools based on sound theory even under approximation may still perform much better.

## Graphical abstract

A vast amount of ecological knowledge generated over the past two decades has hinged upon the ability of model selection methods to discriminate among various ecological hypotheses. We focus on a specific class of competing Bayesian spatial capture recapture (SCR) models and apply and evaluate some of the recommended Bayesian model selection tools: (1) Bayes Factor—using (a) Gelfand‐Dey (b) harmonic mean methods, (2) Deviance Information Criterion, (3) Watanabe‐Akaike's Information Criteria and (4) the posterior predictive loss function. Our study highlights the point that although new model selection tools are emerging (e.g., WAIC) in the applied statistics literature, those tools (e.g., Bayes factor) based on sound theory even under approximation may still perform much better.

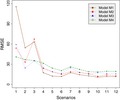

## 1 INTRODUCTION

Following the highly influential text, Burnham and Anderson (2002), on model selection, ecologists and conservation biologists have drastically shifted their inferential practice from the “hypothesis testing” approach to the more appropriate “hypothesis discrimination” approach (Johnson & Omland, 2004). To date, Burnham and Anderson (2002) has been cited 44,168 times (as on January 3, 2019—Google Scholar), demonstrating the impact of this text. One may argue that this contribution has helped increase the pace of growth in ecological knowledge because it has paved the way for researchers to draw inferences more robustly because of the ability to now assess the influence of various competing a priori hypotheses (models) without altering the study question to suit the restrictive hypothesis testing paradigm (Bolker, 2008).

Since a large amount of ecological data are based on field observations, it called for ecologists to take the approach of “detectives” rather than “hypothetico‐deductive” scientists by formulating models using likelihood functions to confront various a priori hypotheses using observational data (Hilborn & Mangel, 1997). And by maximizing the likelihood and using a model selection tool, such as the Akaike's information criterion (Burnham & Anderson, 2002), researchers found a way to place increased faith on models favored by such criteria. Thus, a vast amount of ecological knowledge generated has relied on the robustness of such model selection tools in accurately discriminating hypotheses.

Recently, there has been an increased use of hierarchical models in ecology since they appear to address two important issues: (a) ecological scales are naturally hierarchical in structure and (b) hierarchical models form a natural way of incorporating the observation process (Royle & Dorazio, 2008). With powerful tools such as the MCMC, it is now possible to confront complex ecological models with data in a Bayesian inferential framework (Bolker, 2008). However, it remains unclear as to how to discriminate among competing hypotheses (models) because popular model selection criteria (such as AIC, BIC, or DIC; Burnham & Anderson, 2002) are not easy to apply or work poorly for complex hierarchical models (Millar, 2009).

It is well known that, asymptotically, the Bayes factor is the preferred model selection tool due to its consistency property, that is, to identify the true data‐generating model if (and only if) the true data‐generating model is included in the model set and if the data tend to the limit of infinite informativeness (Ghosh, Delampady, & Samanta, 2006; Robert, 2007). However, this property holds only under certain regularity conditions that are often difficult to verify for complex models (Berger, Ghosh, & Mukhopadhyay, 2003; Dass & Lee, 2004; Ghosh & Samanta, 2001). More prominently, there is vast literature expressing the difficulties in computing the marginal likelihood in applied statistical problems (Chan & Eisenstat, 2015; Wang, 2018). Hence, it becomes necessary to also consider alternatives to the Bayes factor or to find novel ways of applying them in practice.

Recently, Hooten and Hobbs (2015) summarized a wide array of Bayesian model selection methods that are available to ecologists. However, the generality of the recommendations provided by them remains unknown. Given such innate difficulties involved in discovering the “ideal” model selection tool both from the standpoint of theory *and* its application to a broad class of models, it appears to be prudent to explore the model selection issue by conditioning, at least, on a particular *class* of models.

Here, we evaluate various Bayesian model selection tools on a class of Bayesian spatial capture–recapture (SCR) models that are now used frequently for animal density estimation (Royle, Chandler, Sollmann, & Gardner, 2013). Although, previously, Goldberg et al. (2015) has attempted to apply the Bayes Factor (Gelfand‐Dey estimator) in an abundance estimation problem for leopards (*Panthera pardus*), their approach of computing the ratio term in the estimator seems inaccurate in the context of how the denominator has to be computed according to Gelfand and Dey (1994). Thus, we evaluate various Bayesian model selection tools by: (a) defining a class of competing models (in our case, these include the model developed in Dey, Delampady, Karanth, and Gopalaswamy (2019) along with simplified alternatives) that vary both in terms of structural and model complexity (b) simulating data sets from a “true” model (c) practically implementing a variety of Bayesian model selection tools, and in specific cases, also proposing alternatives previously not defined (d) assessing the efficacy of these implementations from the standpoint of model selection and parameter estimation and (e) providing recommendations to practitioners based on our results.

## 2 METHODS

We describe here the sampling design and development of the competing models in the candidate model set.

### 2.1 The candidate model set

Capture–recapture surveys are conducted by placing an array of detectors (e.g., camera traps, hair snares) to sample the species of interest within a bounded region over a fixed period of time. As an extension, spatial capture–recapture (SCR) models draw inference on the spatial distribution of animals using their spatial locations from the recorded capture–recapture samples (Royle et al., 2013).

In photographic capture–recapture surveys, an array of camera traps are placed over the study area. Each camera trap station consists of two cameras placed opposite to one another to photograph the flanks of animals passing by. Naturally marked species can be identified by the unique patterns on their flanks. However, an animal passing through a camera trap station does not necessarily result in identifiable flank photographs from both the sides. This is because there are many known and unknown factors that can influence camera firing rates. Such a detection process results in uncertain or partially identified individuals in the spatial capture—recapture sample and thus provided the motivation for the development of Dey et al. (2019). We summarize the description of the model below.

#### 2.1.1 Sampling situation

The notation used in this article is described in Tables 1 and 2. However, we describe a few variables and parameters for ease in the model description below. Consider a capture–recapture survey of a species with naturally marked individuals in which two detectors are collocated at *J* trap stations (within a bounded geographic region $V\subset R^{2}$) and kept active for *K* sampling occasions. An individual can be completely identified if both the detectors record the individual simultaneously at least once during the course of study (Royle, 2015). We assume that each detector captures some mutually exclusive attributes of an individual. These capture outcomes are recorded as binary observations $y_{\mathrm{ijk}}^{\left(1\right)}$ and $y_{\mathrm{ijk}}^{\left(2\right)}$ for an individual *i* at trap station **x** *_j_* on sampling occasion *k* corresponding to detectors 1 and 2, respectively. The paired Bernoulli outcomes $y_{\mathrm{ijk}}=\left(y_{\mathrm{ijk}}^{\left(1\right)},\;y_{\mathrm{ijk}}^{\left(2\right)}\right)$ give rise to bilateral spatial capture–recapture data for each individual *i* at location **x** *_j_* on occasion *k*. The array of a bilateral capture history for an individual *i* is denoted by $Y_{i,\mathrm{obs}}=\left(Y_{i,\mathrm{obs}}^{\left(1\right)},\;Y_{i,\mathrm{obs}}^{\left(2\right)}\right)={\left(\left(y_{\mathrm{ijk}}^{\left(1\right)},\;y_{\mathrm{ijk}}^{\left(2\right)}\right)\right)}_{j,k}$, which is of dimension 2 × *J* × *K*. In Example 2.1, we provide an example of a sample data set coming out of a spatial capture–recapture survey with two detectors deployed at each station.

**Table 1:** Notations of variables and parameters used in this article

Variables and parameters	Definition
V	A bounded geographic region of scientific or operational relevance where a population of individuals of certain species reside
*N ~ *Binomial (*M, ψ*)	Population size of the superpopulation, that is, the number of individuals within V
*M*	Maximum number of individuals within the state space V This is a fixed quantity defined by the investigator
*ψ*	Proportion of individuals that are real and present within V
*θ*	Probability that an individual is male
*J*	Number of trap stations in V
*K*	Number of sampling occasions
*R*	Maximum permissible value of movement range for each individual during the survey
*ω*_0_	Baseline trap entry probability in the models *M*_1_ and *M*_2_, that is, probability that an individual passes through a trap station assuming its center of activity is also located at that trap station
*p*_0_	Baseline detection probability in the models *M*_2_ and *M*_4_, that is, probability that an individual is detected by a detector assuming its center of activity is also located at that trap station
*σ*	*σ* measures the spatial extent of movement around individual activity center. *σ* = *σ_m_* for male individuals, *σ* = *σ_f_* for female individuals
$d_{\mathrm{ij}}=d\;\left(s_{i},\;x_{j}\right)=\;∥s_{i}-x_{j}\;∥$	Euclidean distance between points **s** *_i_* and **x** *_j_*
$\eta_{j}\left(s_{i},u_{i}\right)=\;\omega_{0}\exp\left(-\frac{d{\left(s_{i},x_{j}\right)}^{2}}{2\sigma {\left(u_{i}\right)}^{2}}\right)$	Probability that an individual *i* passes through a trap station **x** *_j_* on some occasion *k* and *σ* is modeled as a function of individual covariate on sex category *u_i_*
$\eta_{j}\left(s_{i}\right)=\;\omega_{0}\exp\left(-\frac{d{\left(s_{i},x_{j}\right)}^{2}}{2\sigma^{2}}\right)$	Probability that an individual *i* passes through a trap station **x** *_j_* on some occasion *k*
*ϕ*	Probability that an individual *i* is detected by a detector on some occasion *k* given that it is present at that trap

Notations pertaining to model selection tools	Definition
${\hat{m}}_{\text{GD}}\left(Y\right)$	Gelfand‐Dey estimator of the marginal likelihood of data *m*(**Y**)
${\hat{m}}_{\text{HM}}\left(Y\right)$	Harmonic mean estimator of the marginal likelihood of data *m*(**Y**)
*p*_DIC_	Correction term for bias due to overfitting in DIC criterion
*p*_WAIC_	Correction term for bias due to overfitting in WAIC criterion
*D*_∞_	Posterior predictive loss criterion

Note

Bold symbols represent collections (vectors).

**Table 2:** Notations of latent variables and data used in this article

Latent variables	Definition
**S**	Locations of the activity centers of *N* animals within V
**s** *_i_* = (*s_i_*_1_, *s*_i2_)′	Location of individual *i*'s activity center.
**Z** = (*z*_1_, *z*_2_, …, *z_M_*)′	A vector of Bernoulli variables, *z*_i_ = 1 if individual *i* is present
**u** = (u_1_, …, *u_M_*)′	A vector of Bernoulli variables, *u_i_* = 1 if individual *i* is male in the population and *u_i_* = 0 if it is a female
$u_{0}\;\left(\subset u\right)$	Vector of “missing” binary observations on sexes of the list of *M* individuals
**L**=(*L*_1_, *L*_2_, …, *L_M_*)′	Each *L_i_* takes value in {1,2,…,*M*} and denotes the true index of *i*th detector 2 individual

Data	Definition
**x** *_j_* = (*x_j_*_1_, *x_j_*_2_)′	Location of *j*th trap station for detectors
$u_{\mathrm{obs}}\;\left(\subset u\right)$	Vector of “recorded” binary observations on sexes of the captured individuals
$y_{\mathrm{ijk}}^{\left(1\right)}$	$y_{\mathrm{ijk}}^{\left(1\right)}=1$ if individual *i* is detected in detector 1 at trap station **x** *_j_* on occasion *k*, $y_{\mathrm{ijk}}^{\left(1\right)}=0$ if not detected in detector 1
$y_{i\cdot \cdot }^{\left(1\right)}=\sum_{j=1}^{J}\sum_{k=1}^{K}y_{\mathrm{ijk}}^{\left(1\right)}$	Number of times individual *i* got detected in detector 1 over *J* trap stations and *K* occasions
$y_{\mathrm{ijk}}^{\left(2\right)}$	$y_{\mathrm{ijk}}^{\left(2\right)}=1$ if individual *i* is detected in detector 2 at trap station **x** *_j_* on occasion *k*, $y_{\mathrm{ijk}}^{\left(2\right)}=0$ if not detected in detector 2
$y_{i\cdot \cdot }^{\left(2\right)}=\sum_{j=1}^{J}\sum_{k=1}^{K}y_{\mathrm{ijk}}^{\left(2\right)}$	Number of times individual *i* got detected in detector 2 over *J* trap stations and *K* occasions
*n*	Number of fully identified individuals, each of them is captured by both the detectors on at least one occasion
$Y_{\mathrm{obs}}^{\left(1\right)}=\left(\left(y_{\mathrm{ijk}}^{\left(1\right)}\right)\right)$	Array of individual‐specific capture histories obtained by detector 1 (dimension *n* × *J* × *K*)
$Y_{\mathrm{obs}}^{\left(2\right)}=\left(\left(y_{\mathrm{ijk}}^{\left(2\right)}\right)\right)$	Array of individual‐specific capture histories obtained by & detector 2 (dimension *n* × *J* × *K*)
**Y** ^(^ **^1^** ^)^	Zero augmented array of individual‐specific capture histories corresponding to detector 1 (dimension *M* × *J* × *K*)
**Y** ^(^ **^2^** ^)^	Zero augmented array of individual‐specific capture histories corresponding to detector 2 (dimension *M* × *J* × *K*)
**Y** ^(^ **^2^** ^*)^	Reordered **Y** ^(^ **^2^** ^)^ according to **L** (dimension *M* × *J* × *K*)
$n_{\mathrm{ij}}=\sum_{k=1}^{K}I\left(y_{\mathrm{ijk}}^{\left(1\right)}+y_{\mathrm{ijk}}^{\left(2\right)}>0\right)$	Number of times individual *i* got detected at trap *j* on at least one of its sides over *K* occasions
$n_{i\cdot }=\sum_{j=1}^{J}n_{\mathrm{ij}}$	Number of times individual *i* got detected on at least one of its sides over *J* traps and *K* occasions

Note

Bold symbols represent collections (vectors).

Example 2.1 Suppose a capture–recapture survey is conducted where a pair of detectors (1 and 2) are deployed at each of the 3 ( = *J*) trap stations and kept active for 4 ( = *K*) sampling occasions. Two individuals get fully identified based on their obtained capture histories (captured in both cameras at least once during the survey). The capture history for each of these two fully identified individuals is of dimension 2 × 3 × 4. The detection histories are tabulated in Table 3. Here, individual 1 is fully identified owing to the capture event at trap 2 on occasion 4. Individual 1 is also fully identified as it is captured at trap 2 on occasion 4. We note that in practical applications, full identity of individuals is obtained only when there are metadata, such as time of capture, to ascertain simultaneous captures. Due to the absence of simultaneous capture events in the detection histories of the partially identified individuals, we are uncertain about whether these histories correspond to two different individuals or to the same individual.

**Table 3:** An example of detection histories for two fully identified individuals and partially identified individuals is presented. The circled 1s indicate the simultaneous captures of an individual by the detectors 1 and 2

	Occasion trap	Detectors 1				Occasion trap	Detectors 2			
		1	2	3	4		1	2	3	4
Fully identified individual 1	1	0	1	0	1	1	0	0	1	0
	2	1	0	0	①	2	0	0	0	①
	3	0	0	1	1	3	1	0	0	0
Fully identified individual 2	1	①	0	0	0	1	①	1	0	0
	2	0	0	0	1	2	0	0	0	0
	3	1	1	0	0	3	0	0	1	0
Partially identified individual	1	1	0	0	1	1	—	—	—	—
	2	0	0	1	0	2	—	—	—	—
	3	0	0	0	0	3	—	—	—	—
Partially identified individual	1	—	—	—	—	1	0	0	1	0
	2	—	—	—	—	2	1	0	0	0
	3	—	—	—	—	3	0	0	1	0

#### 2.1.2 Model likelihoods

We have considered four models, denoted by *M*_1_, *M*_2_, *M*_3_, and *M*_4_, and the model likelihoods of the corresponding models follow in Equations ((1), (2), (3), (4)). Each of the four models *M*_1_–*M*_4_ are SECR models for partially identified capture–recapture history. The basic difference between *M*_3_ and *M*_4_ is that *M*_4_ does not explain the latent hierarchy of the two events: trap entry of an animal and subsequent detection conditional on trap entry, whereas the former model *M*_3_ does. *M*_1_ uses the covariate information on sex category and allows the movement scale parameter (*σ* in our case) of *M*_3_ to be gender specific. The differences between *M*_2_ and *M*_4_ are exactly the same (see Table 4). We describe the specific models in below.

**Table 4:** Specification differences in the four competing models

Model	Trap entry and detection parameter separated?	Sex‐specific *σ* (with sex covariate **u**)?
*M*_1_	Yes	Yes
*M*_2_	No	Yes
*M*_3_	Yes	No
*M*_4_	No	No

When detection rates in recorded samples are low due to failure or malfunction of detectors, capture–recapture data may be comprised of individuals with uncertain identities or “partially identified individuals.” Dey et al. (2019) separately accounts for the process of animal arrival within the detection region of a detector and detection process by conditioning on animal arrival—thus modeling the underlying mechanism by which we obtain different events leading to partial identification.

The probability of animal arrival *η_j_* (**s** *_i_*) (termed as “trap entry probability”) is modeled as a decreasing function of Euclidean distance $d\left(s_{i},x_{j}\right)=∥s_{i}-x_{j}∥$ between individual activity center **s** *_i_* and trap station **x** *_j_*: $\eta_{j}\left(s_{i}\right)=\omega_{0}\exp\left(-d{\left(s_{i},\;x_{j}\right)}^{2}/\left(2\sigma^{2}\right)\right)$. Here, *ω*_0_ is regarded as the “baseline trap entry probability” and *σ* quantifies the rate of decline in trap entry probability as *d*(**s** *_i_*, **x** *_j_*) increases. The observation process is parameterized in terms of detection probability *ϕ* which denotes the probability that any arbitrary individual *i* is detected by a detector on some occasion *k* given its arrival at that trap.

The obtained capture history observations $Y_{\mathrm{obs}}^{\left(1\right)}={\left(\left(y_{\mathrm{ijk}}^{\left(1\right)}\right)\right)}_{i,j,k}$ and $Y_{\mathrm{obs}}^{\left(2\right)}={\left(\left(y_{\mathrm{ijk}}^{\left(2\right)}\right)\right)}_{i,j,k}$ from the two detectors 1 and 2 during a spatial capture–recapture survey may not be synchronized as detectors often perform imperfectly. These two observed data arrays are then augmented with “all‐zero” capture histories. We denote the zero‐augmented data sets by **Y** ^(^ **^1^** ^)^ and **Y** ^(^ **^2^** ^)^; each of them is of dimension *M* × *J* × *K*, *M* being an upper bound of the population size. This also makes the dimension of the likelihood fixed in each iteration of the Markov Chain Monte Carlo algorithm which in turn eases computation. A vector of *M* latent binary variables *z* = (*z*_1_,…,*z_M_*)′ is introduced where *z_i_* = 1 implies that individual *i* is a member of the population. We assume that each *z_i_* is a Bernoulli random variable with parameter *ψ* and is independent of other *z_j_*'s. Here *ψ* is the proportion of individuals that are real and present within V. Thus, the true population size *N* follows the Binomial distribution with parameters *M* and *ψ*. The individuals from the two lists obtained from detector 1 and detector 2, respectively, are linked probabilistically by introducing a latent identity variable **L** = (*L*_1_, *L*_2_,…, *L_M_*)′ which is a one‐to‐one mapping from an index set of individuals captured by detector 2 to {1, 2,…,M} giving the true index of each of detector 2 individuals. Without loss of generality, the true identity of each individual in the population is defined to be in the row order of the capture histories of detector 1. Then, the rows of detector 2 data set **Y^(2)^** are reordered as indicated by **L** to synchronize with the individuals of the detector 1 data set **Y^(1)^**. We denote this newly ordered detector 2 data set as Y^(2*)^.

It is sometimes helpful to introduce a binary covariate on sex category *u* on spatial animal movement, *σ*, as in Sollmann et al. (2011). We define *σ* as a function of the latent structural vector $u={\left(u_{1},u_{2},\ldots ,u_{M}\right)}^{\prime }$: $\sigma \left(u_{i}\right)=\sigma_{m}$, if *u_i_ *= 1, that is, individual *i* is a male; *σ*(*u_i_*) = *σ_f_*, if *u*_i_ = 0; that is, individual *i* is a female. *u*_i_'s are independently and identically distributed Bernoulli random variables with parameter *θ*, *θ* being the probability that an arbitrary individual in the population is male. Let $u_{\mathrm{obs}}\left(\subset u\right)$ be a vector of binary observations on sex category of the captured individuals. The vector of latent missing observations in **u** is denoted by **u**_0_. Assuming that covariate information on individual sex category is available, the joint density of **Y** ^*^ and **u** under Dey et al. (2019) is the following:

(1) $$\begin{array}{c} f(Y^{*},u_{\mathrm{obs}}|\theta ,\;\phi ,\;\omega_{0},\;\sigma_{m},\;\sigma_{f},\;u_{0},\;z,\;S,\;L)\\ =\prod_{i=1}^{M}\left[{\left\{\begin{array}{c} \theta^{u_{i}}{\left(1-\theta \right)}^{1-u_{i}}\phi^{y_{i\cdot \cdot }}{\left(1-\phi \right)}^{2n_{i\cdot }-y_{i\cdot \cdot }}\prod_{j=1}^{J}\eta_{j}{\left(s_{i},\;u_{i}\right)}^{n_{\mathrm{ij}}}\\ {\left\{\left(1-\eta_{j}\left(s_{i},\;u_{i}\right)\right)+\eta_{j}\left(s_{i},\;u_{i}\right){\left(1-\phi \right)}^{2}\right\}}^{K-n_{\mathrm{ij}}} \end{array}\right\}}^{z_{i}}\right], \end{array}$$

where $\eta_{j}\left(s_{i},u_{i}\right)=\;\omega_{0}\exp\left(-d{\left(s_{i},x_{j}\right)}^{2}/\left(2\sigma {\left(u_{i}\right)}^{2}\right)\right)$ denotes the probability that an individual *i* passes through a trap station **x** *_j_* on some occasion *k*, $y_{i\cdot \cdot }=y_{i\cdot \cdot }^{\left(1\right)}+y_{I\cdot \cdot }^{\left(2*\right)},y_{i\cdot \cdot }^{\left(1\right)}=\sum_{j=1}^{J}\sum_{k=1}^{K}y_{\mathrm{ijk}}^{\left(1\right)},y_{i\cdot \cdot }^{\left(2\right)}=\sum_{j=1}^{J}\sum_{k=1}^{K}y_{\mathrm{ijk}}^{\left(2\right)},n_{\mathrm{ij}}=\sum_{k=1}^{K}I\left(y_{\mathrm{ijk}}^{\left(1\right)}+y_{\mathrm{ijk}}^{\left(2*\right)}>0\right)$ is the number of times individual *i* got detected on at least one the detectors over *K* occasions and $n_{i\cdot }=\sum_{j=1}^{J}n_{\mathrm{ij}}$. The above Equation (1) can be regarded as zero‐inflated Bernoulli density with extra zeros coming from no trap entry. Prior to Dey et al. (2019), Royle (2015) proposed an SCR model for partially identified individuals coming from spatial capture–recapture surveys. The joint density of **Y** ^*^ and **u** under Royle (2015) is the following:

(2) $$\begin{array}{c} f_{R}\left(Y^{*},u_{\mathrm{obs}}|\theta ,\;p_{0},\;\sigma_{m},\;\sigma_{f},\;u_{0},\;z,\;S,\;L\right)=\\ \prod_{i=1}^{M}\left[{\left\{\theta^{u_{i}}{\left(1-\theta \right)}^{1-u_{i}}\prod_{j=1}^{J}p_{j}{\left(s_{i},\;u_{i}\right)}^{y_{ij\cdot }}{\left(1-p_{j}\left(s_{i},\;u_{i}\right)\right)}^{2K-y_{ij\cdot }}\right\}}^{z_{i}}\right], \end{array}$$

where *p_j_* (**s** *_i_*, *u*_i_) = *p*_0_ exp(−d(**s**_i_, **x** *_j_*)^2^/(2*σ*(*u_i_*)^2^)) denotes the probability that an individual *i* is detected at **x** *_j_* on occasion *k*. Note that, unlike model (Equation 1), here movement through the detection region is considered inherently as a part of the observation process and *p*_0_ is regarded as “baseline detection probability” and *σ*, although related to animal movement, is regarded as the *rate of decline in detection probability*. Qualitatively, the absence of *ϕ* in (Equation 2) distinguishes this model from (Equation 1) and can be regarded as a less general model. Recently, Augustine et al. (2018) extended Royle (2015) by introducing separate parameters to distinguish captures by both detectors and captures by only one of the detectors.

In the absence of the sex covariate **u**, the joint density of $Y^{*}:\;=\left(Y^{\left(1\right)},Y^{\left(2*\right)}\right)=\left(\left(y_{\mathrm{ijk}}^{\left(1\right)},y_{\mathrm{ijk}}^{\left(2*\right)}\right)\right)$ under Dey et al. (2019) and Royle (2015) is, respectively, as given below:

(3) $$\begin{array}{c} f(Y^{*}|\phi ,\omega_{0},\sigma ,z,S,L)=\\ \prod_{i=1}^{M}{\left\{\phi^{y_{i\cdot \cdot }}{\left(1-\phi \right)}^{2n_{i\cdot }-y_{i\cdot \cdot }}\prod_{j=1}^{J}\eta_{j}{\left(s_{i}\right)}^{n_{\mathrm{ij}}}{\left\{\left(1-\eta_{j}\left(s_{i}\right)\right)+\eta_{j}\left(s_{i}\right){\left(1-\phi \right)}^{2}\right\}}^{K-n_{\mathrm{ij}}}\right\}}^{z_{i}}, \end{array}$$

(4) $$f_{R}\left(Y^{*}|p_{0},\sigma ,z,S,L\right)=\prod_{i=1}^{M}{\prod_{j=1}^{J}\left\{p_{j}{\left(s_{i}\right)}^{y_{ij\cdot }}{\left(1-p_{j}\left(s_{i}\right)\right)}^{2K-y_{ij\cdot }}\right\}}^{z_{i}},$$

where $y_{i\cdot \cdot }=y_{i\cdot \cdot }^{\left(1\right)}+y_{i\cdot \cdot }^{\left(2*\right)},\eta_{j}\left(s_{i}\right)=\;\omega_{0}\exp\left(-d{\left(s_{i},x_{j}\right)}^{2}/\left(2\sigma^{2}\right)\right)$ denotes the probability that an individual *i* passes through a trap station **x** *_j_* on some occasion *k* and *p_j_*(**s** *_i_*) = *p*_0_ exp(−d(**s**_i_, **x** *_j_*)_2_/(2*σ* ^2^)) denotes the probability that an individual *i* is detected at a trap station **x** *_j_* on some occasion *k*.

The assumed prior distributions for the model parameters are as follows: a uniform distribution over the interval (0,1) for the probability parameters *ϕ*, *ω*_0_, *p*_0_, *ψ*, and *θ*; a uniform distribution over the interval (0,*R*) for parameters *σ*, *σ_m_*, and *σ_f_* where *R* is high enough to expect that it would be impossible for animals to exhibit movement as widely as this scale during sampling. The prior distributions for these model parameters *ϕ*, *ω*_0_, *p*_0_, *ψ*, and *θ*, *σ*, *σ_m_* are assumed to be independent. To ensure that the marginal distribution of the data is well defined, we have assumed proper priors for each of these parameters (Gopalaswamy & Delampady, 2016). We assume a uniform prior over the entire state space V for each location of the activity center **s** *_i_* and that these **s** *_i_*'s are independently distributed. The latent variable **L** is assumed to have a uniform prior distribution over the permutation space of {1, …, *M*}. The prior specifications remain the same for all the model fits. The MCMC algorithm used to sample from the respective posterior density under each model is detailed in Appendix S1D. Thus, we have the four models,

*M*_1_: Model with density (Equation 1),
*M*_2_: Model with density (Equation 2),
*M*_3_: Model with density (Equation 3), and
*M*_4_: Model with density (Equation 4).

Model selection tools are used to find the “best” fitted model among a set of competing models. To provide an understanding of what such a best model is, Shmueli (2010) discusses two broad modelling philosophies—explanatory and predictive. Explanatory modeling of the data set is only relevant to obtain the most accurate representation of the underlying theory, whereas predictive modelling seeks to minimize the combination of bias and estimation variance. Hence, a model that explains the data well may not have the best predictive ability and vice versa. This naturally leads to two performance aspects by which a model can be assessed—(a) how well does the model explain the observed data set, that is, to what degree of confidence can it be thought of as a model from which the data generated, (b) how good is its predictive ability.

On such criteria, a wide range of model selection tools have thus emerged in the applied statistical literature (see Gelman, Hwang, & Vehtari, 2014; Höge, Guthke, & Nowak, 2019; Höge, Wöhling, & Nowak, 2018; Hooten & Hobbs, 2015; Kass & Raftery, 1995; for examples). In all these cases, however, we are assuming that the “true” model is in the list of competing models. Such classes of problems are formally categorized as ‘M‐closed’ (Bernardo & Smith, 1994; Clarke, Clarke, & Yu, 2013; Vehtari & Ojanen, 2012) and is generally considered to be well‐studied (Clarke, Clarke, & Yu, 2013).

However, for the practitioner, the applicability of such guidelines for model selection is not clear because often such guidelines are formed using specific examples (Hooten & Hobbs, 2015) or based on asymptotic arguments in a theoretic sense (for example, Shibata, 1989; Stone, 1977; Watanabe, 2010). Even when the purpose of model selection is clear (explanatory or predictive) and also the model selection criterion is fixed, what techniques practically work is not clear. Practitioners often wish to confront fairly complex models (e.g., hierarchical models) with their data, and it is often a challenge to directly apply a candidate set of model selection tools without incorporating some approximations. Given these approximations due to model complexity and with limited sample sizes, the validity of such general guidelines (Hooten & Hobbs, 2015) remains to be tested in practice. Therefore, in this study, we apply a range of model selection tools and test the validity of such general model selection guidelines in the context of a popular class of models, spatial capture—recapture models, used for estimating animal density of some of the world's most iconic species (Broekhuis & Gopalaswamy, 2016; Elliot & Gopalaswamy, 2017; Royle, Karanth, Gopalaswamy, & Kumar, 2009; Sollmann et al., 2011).

### 2.2 Candidate model selection tools

We have considered four different Bayesian model selection methods for application and evaluation: *Bayes factors*, *Deviance Information Criterion* (DIC), *Watanabe‐Akaike information criterion* (WAIC), and *posterior predictive loss*. Two popular model selection tools (AIC and BIC; Burnham & Anderson, 2002) are not used here because they impose restrictive assumptions on the parameter space as the sample size increases—situations often encountered in many hierarchical models (Royle & Dorazio, 2008). For example, in the SCR models we study here, the concept of “number of parameters” is unclear and we therefore cannot apply criteria such as the AIC and the BIC directly.

We clarify our aim here is to discriminate between hypotheses (models) and not to conduct hypothesis tests of the model parameters. In the hypothesis testing paradigm that applies in our setup, it usually requires for models to be nested so that statistical tests (such as likelihood ratio tests) between different parameter values can be performed. However, this limitation does not apply to model selection under the hypothesis discrimination paradigm.

#### 2.2.1 Bayes factors

Model comparison using Bayes factors requires the computation of the marginal likelihood, which involves the integration $m\left(Y|M_{i}\right)=\int f\left(Y|\mu ,M_{i}\right)\pi \left(\mu \right)d\mu$ where $f(Y|\mu ,M_{i})$ denotes the model likelihood and π(**µ**) denotes the prior density of the parameters **µ** under *M_i_*. This integration is difficult to compute in practice unless the models are very simple in structure, which is often not the case in ecology. Therefore, computation of marginal likelihood continues to be an active area of research in Statistics (Wang & Meng, 2016; Wang et al., 2018).

##### Estimation of marginal likelihood of data

Under our model settings, **Y** = (**Y** ^*^, **u_obs_**) for models *M*_1_, *M*_2_, and **Y** = **Y** ^*^ for the other models *M*_3_, *M*_4_. **µ** denotes the collection of all parameters and latent variables for each model as a generic notation. Specifically, let **µ** = (**µ** *_p_*, **µ**_s_), where **µ** *_p_* is the collection of scalar parameters and **µ** *_s_* is the collection of all latent variables. The Gelfand‐Dey estimator of marginal likelihood of data m(**Y**) is expressed as:

(5) $${\hat{m}}_{\text{GD}}\left(Y\right)={\left[\frac{1}{N_{\mathrm{iter}}}\sum_{d=1}^{N_{\mathrm{iter}}}\frac{g(\mu^{\left(d\right)})}{f\left(Y|\mu^{\left(d\right)}\right)\pi \left(\mu^{\left(d\right)}\right)}\right]}^{-1},$$

where {**µ** ^(^ *^d^* ^)^: *d* = 1, …, *N*_iter_} is a set of MCMC draws from the posterior π(**µ**|**Y**) and *g*(**µ**) is a tuning density. It is to be noted that by specifying *g*(**µ**) = π(**µ**) in (Equation 5), we obtain the harmonic mean estimator of the marginal likelihood

(6) $${\hat{m}}_{\text{HM}}\left(Y\right)={\left[\frac{1}{N_{\text{iter}}}\sum_{d=1}^{N_{\text{iter}}}\frac{1}{f(Y|\mu^{\left(d\right)})}\;\right]}^{-1}.$$

Details on these estimators and their properties can be found in Gelfand and Dey (1994), Kass and Raftery (1995). The harmonic mean estimator is easy to compute by just calculating the model likelihood at each MCMC sample draws from the posterior distribution. Although it is known that the harmonic mean estimator is a consistent estimator of the marginal likelihood, as noted in Kass and Raftery (1995) and Newton and Raftery (1994), it can have a simulation pseudo‐bias (Lenk, 2009). This bias occurs due to the limitations in numerical computations. Indeed, our study here aims to assess how and whether these potential biases in computation influence the model selection process for the particular class of models we consider—partial identity SCR models, in our case.

For our problem, the computation of (Equation 5) requires us to obtain the integrated likelihoods (marginals) under the different models that we consider. This becomes particularly tricky in the presence of high‐dimensional latent variables such as **u**_0_, **z**, **S**, and **L**, which are elements of **µ** *_s_*.

We have developed a novel approach to compute an estimate of the marginal likelihood. Here we partition the parameters of a model into two sets (real‐valued scalar and high‐dimensional structural parameters, which are denoted by **µ** *_p_* and **µ** *_s_*, respectively) and then propose two approximating strategies to account for the high‐dimensional structural parameters, ${\hat{\mu }}_{s}$, before applying the Gelfand‐Dey estimator. This general approach of partitioning can be applied to many other classes of hierarchical models as well and not limited to SCR models.

We describe below two *approximating* approaches to compute the Gelfand‐Dey estimator: the maximum a posteriori (MAP) approximation approach and the integrated likelihood (IL) approach.

##### Approach 1: MAP approximation

In this approach, we fix the high‐dimensional variables at their MAP estimates ${\hat{\mu }}_{s}$, assuming that their posterior distributions are well summarized by these estimates, which are derived from the MCMC draws. The Gelfand‐Dey estimator is then computed using the formula,

(7) $${\hat{m}}_{\text{GD}}\left(Y\right)={\left[\frac{1}{N_{\text{iter}}}\sum_{d=1}^{N_{\text{iter}}}\frac{g(\mu_{p}^{\left(d\right)})}{f\left(Y|\mu_{p}^{\left(d\right)},{\hat{\mu }}_{s}\right)\pi \left(\mu_{p}^{\left(d\right)}\right)}\right]}^{-1}.$$

where $\left\{\left(\mu_{p}^{\left(d\right)},\;\mu_{s}^{\left(d\right)}\right):\;d=1,\;\ldots ,\;N_{\text{iter}}\right\}$ is a set of MCMC draws from the posterior π(**µ** *_p_*, **µ** *_s_*|**Y**). We begin with $\left(\mu_{p}^{\left(d_{0}\right)},\;\mu_{s}^{\left(d_{0}\right)}\right)$ as an initial estimate of (**µ*_p_***, **µ*_s_***) where $f\left(Y|\mu_{p}^{\left(d_{0}\right)},\;\mu_{s}^{\left(d_{0}\right)}\right)\pi \left(\mu_{p}^{\left(d_{0}\right)},\;\mu_{s}^{\left(d_{0}\right)}\right)=\underset{d}{\text{max}}\left\{f\left(Y|\mu_{p}^{\left(d\right)},\;\mu_{s}^{\left(d\right)}\right)\pi \left(\mu_{p}^{\left(d\right)},\;\mu_{s}^{\left(d\right)}\right)\right\}.$

This estimate of the posterior mode of (**µ_p_**, **µ_s_**) may not be optimal since in our high‐dimensional parameter setting, an MCMC sample of a practical size may not be enough to extensively explore the posterior surface. We, therefore, fix one of the parameters $\mu_{s}=\mu_{s}^{\left(d_{0}\right)}$ and explore the posterior surface to find *d*_1_ such that $f\left(Y|\mu_{p}^{\left(d_{1}\right)},\;\mu_{s}^{\left(d_{0}\right)}\right)\pi \left(\mu_{p}^{\left(d_{1}\right)},\;\mu_{s}^{\left(d_{0}\right)}\right)=\underset{d}{\text{max}}\left\{f\left(Y|\mu_{p}^{\left(d\right)},\;\mu_{s}^{\left(d_{0}\right)}\right)\pi \left(\mu_{p}^{\left(d\right)},\;\mu_{s}^{\left(d_{0}\right)}\right)\right\}$. In this way, we obtain an improved MAP estimate of (**µ** *_p_*, **µ** *_s_*), $\left(\mu_{p}^{\left(d_{1}\right)},\;\mu_{s}^{\left(d_{0}\right)}\right)$, if $f\left(Y|\mu_{p}^{\left(d_{1}\right)},\mu_{s}^{\left(d_{0}\right)}\right)\pi \left(\mu_{p}^{\left(d_{1}\right)},\mu_{s}^{\left(d_{0}\right)}\right)>f\left(Y|\mu_{p}^{\left(d_{0}\right)},\mu_{s}^{\left(d_{0}\right)}\right)\pi \left(\mu_{p}^{\left(d_{0}\right)},\mu_{s}^{\left(d_{0}\right)}\right).$

Similarly, we then fix $\mu_{p}=\mu_{p}^{\left(d_{1}\right)}$ and find $\mu_{s}^{\left(d_{2}\right)}$. This procedure is continued iteratively to eventually give us the best MAP estimate of the posterior mode $\left({\hat{\mu }}_{p},\;{\hat{\mu }}_{s}\right)$. Suitable transformations of the parameters ensure that all the points in $\left\{\left(\mu_{p}^{\left(a\right)},\;\mu_{s}^{\left(b\right)}\right):\;a,b=1,\;\ldots ,\;N_{\text{iter}};\;a\neq b\right\}$ belong to the posterior support. A single MCMC chain is to be used for the algorithm, and we have shown that the obtained MAP estimate using our algorithm will be better than the usual estimate (**µ** *_p_*, **µ** *_s_*) (see Appendix S1A). In theory, we can also increase sample size by merging all the chain outputs after convergence.

##### Approach 2: Integrated likelihood (IL) approximation

Ideally, we would like to compute the marginal likelihood m(**Y**) by integrating out all the latent variables with respect to their corresponding prior distributions from the model likelihoods. However, in the case of model likelihoods (Equations (1), (2), (3), (4)), this integration is computationally difficult for the permutation vector **L** because of its very large support. The integration over the variables **u**_0_ and **z** can be performed analytically. The integration over **S** is evaluated numerically by partitioning the region V into a sufficiently fine grid and then evaluating a Riemann sum (as direct integration cannot be expressed in a closed form). This integrated likelihood can then be used in (Equation 5) for estimating the marginal likelihood

(8) $${\hat{m}}_{\text{GD}}\left(Y\right)={\left[\frac{1}{N_{\mathrm{iter}}}\sum_{d=1}^{N_{\text{iter}}}\frac{g(\mu_{p}^{\left(d\right)},L^{\left(d\right)})}{f\left(Y|\mu_{p}^{\left(d\right)},L^{\left(d\right)}\right)\pi \left(\mu_{p}^{\left(d\right)},L^{\left(d\right)}\right)}\right]}^{-1}.$$

One downside of the IL approximation approach is the lack of clarity about the interdependencies between the latent variables **u**_0_, **z**, **S**, and **L** after carrying out the integrations. The derivations of the integrated likelihoods for each of the four models *M*_1_–*M*_4_ are given in Appendix S1B by ignoring any possible interdependencies. We have also assessed the robustness of the Gelfand‐Dey estimator by computing the marginal likelihood estimates using different tuning densities, for example, multivariate normal density, multivariate‐t density with varying degrees of freedom, and the truncated normal density following the suggestion of Geweke (1999). These technical details are described in Appendix S1C.

#### 2.2.2 Deviance information criterion

Deviance is defined as *D*(**µ**) = −2log f(**Y**|**µ**). The deviance information criterion (DIC) is then defined as $\text{DIC}=D\left(\hat{\mu }\right)+2p_{\text{DIC}}$, where $\hat{\mu }$ is an estimate of **µ**. The term *p*_DIC_ can be viewed as effective number of parameters, which is a bias correction in evaluating a model's predictive accuracy. Two versions of *p*_DIC_ are generally used (Gelman, Carlin, et al., 2014; Spiegelhalter, Best, Carlin, & van der Linde, 2002):

(9) $$\begin{array}{c} p_{\text{DIC1}}=E_{\mu |y}\left[D\left(\mu \right)\right]-D\left(\hat{\mu }\right)=2\left(\log f\left(y|\hat{\mu }\right)-E_{\mu |y}\left[\log f\left(y|\mu \right)\right]\right),\\ p_{\text{DIC}2}=2V{\text{ar}}_{\mu |y}\left[\log f\left(y|\mu \right)\right]. \end{array}$$

Although *p*_DIC1_ has been considered to be numerically more stable, *p*_DIC2_ has the advantage of being always positive (Gelman, Hwang, & Vehtari, 2014). A model with smaller DIC value is preferred. Model comparison using DIC is not invariant to parameterization and depends on the components of the model likelihood to be considered as the likelihood. Spiegelhalter et al. (2002) suggests that practitioners carefully decide on the parameters of interest so that they can avoid this potential pitfall. This advice is often difficult to implement in practice, especially when there exists inherent ambiguity in the interpretation of latent parameters. We note that Celeux, Forbes, Robert, and Titterington (2006) suggests several forms for the DIC that can be used for different hierarchical models but does not recommend any particular form as the best.

For the computation of deviance, we have use the MAP estimate of **µ** to obtain $\hat{\mu }$ instead of the posterior mean, due to the presence of binary latent variables and unknown permutation vectors in the likelihood. We have then computed two versions of *p*_DIC_ (Gelman, Carlin, et al., 2014; Hooten & Hobbs, 2015) using the MCMC draws $\left\{\mu^{\left(d\right)}:d=1,\ldots ,N_{\text{iter}}\right\}$ from π(**µ**|**Y**) as follows:

(10) $$\begin{array}{c} {\hat{p}}_{\text{DIC}1}=2\log f\left(Y|\hat{\mu }\right)-\frac{2}{N_{\text{iter}}}\sum_{d=1}^{N_{\text{iter}}}\log f\left(Y|\mu^{\left(d\right)}\right),\\ {\hat{p}}_{\text{DIC}2}=2\left[\frac{1}{N_{\mathrm{iter}}}\sum_{d=1}^{N_{\text{iter}}}{\left(\log f\left(Y|\mu^{\left(d\right)}\right)-\frac{1}{N_{\mathrm{iter}}}\sum_{d=1}^{N_{\mathrm{iter}}}\log f\left(Y|\mu^{\left(d\right)}\right)\right)}^{2}\right] \end{array}$$

#### 2.2.3 Watanabe‐Akaike information criterion

The Watanabe‐Akaike information criterion (WAIC) is a Bayesian version of the AIC as it uses the posterior predictive distribution of the data to estimate the out‐of‐sample predictive accuracy of the model. Watanabe (2010) introduced the WAIC criterion based on the assumption of independence between the data points and has shown its asymptotic equivalence with cross‐validation. In our model formulations, we have assumed that the different data points correspond to capture–recapture data set for each of the *M* individuals. The WAIC is then defined as $\text{WAIC}=-2\sum_{i=1}^{M}\log E_{\mu |Y}\left(f(Y_{i}|\mu )\right)+2p_{\text{WAIC}}$. A model with smaller WAIC value is preferred. In computing WAIC, we partition data *Y* in terms of individuals (*Y*_1_, *Y*_2_,…, *Y_M_*). We compute the two commonly used versions of *p*_WAIC_ (Hooten & Hobbs, 2015) using MCMC draws{**µ** ^(d)^: *d* = 1,…,*N*_iter_} from π (µ|**Y**) as follows:

(11) $$\begin{array}{c} {\hat{p}}_{\text{WAIC}1}=2\sum_{i=1}^{M}\left\{\log\left(\frac{1}{N_{\text{iter}}}\sum_{d=1}^{N_{\text{iter}}}f(Y_{i}|\mu^{\left(d\right)})\right)-\frac{1}{N_{\text{iter}}}\sum_{d=1}^{N_{\text{iter}}}\log f(Y_{i}|\mu^{\left(d\right)})\right\},\\ {\hat{p}}_{\text{WAIC}2}=\sum_{i=1}^{M}\left\{\frac{1}{N_{\text{iter}}}\sum_{d=1}^{N_{\text{iter}}}{\left(\log f\left(Y_{i}|\mu^{\left(d\right)}\right)-\frac{1}{N_{\text{iter}}}\sum_{d=1}^{N_{\text{iter}}}\log f\left(Y_{i}|\mu^{\left(d\right)}\right)\right)}^{2}\right\}. \end{array}$$

We propose another version for *p*_WAIC_ based on absolute error loss, recognizing that large variability in **µ** ^(^ *^d^* ^)^ and the magnitude of squared errors themselves may have an impact on the efficiency of *p*_WAIC_ of the above forms:

(12) $${\hat{p}}_{\text{WAIC}3}=2\sum_{i=1}^{M}\left\{\frac{1}{N_{\mathrm{iter}}}\sum_{d=1}^{N_{\text{iter}}}\left|\log f\left(Y_{i}|\mu^{\left(d\right)}\right)-\frac{1}{N_{\text{iter}}}\sum_{d=1}^{N_{\text{iter}}}\log f\left(Y_{i}|\mu^{\left(d\right)}\right)\right|\right\}.$$

From a decision‐theoretic perspective, we think it is interesting to compare WAIC2, which is based on a square error loss function with WAIC3 that is based on an absolute error loss function. The comparison based on a simulated data analysis is described later in Section 4.

#### 2.2.4 Posterior predictive loss

Gelfand and Ghosh (1998) derived a model selection criterion, popularly known as the posterior predictive loss criterion, by adopting a decision‐theoretic approach for measuring predictive accuracy of a model. The posterior predictive loss *D_w_* criterion (based on a square error loss function) is composed of squared bias, variance of a new prediction, and a parameter *w*. The parameter *w* indicates the relative weight given to the loss for departure of the new predicted value from the observed data against departure of the same from the new data. In practice, this weight is taken to be very large, and asymptotically as *w* →∞, the *D*_∞_ criterion is obtained as follows under our model setting:

(13) $$D_{\infty }=\sum_{i=1}^{2MJK}{\left(y_{i,\text{vec}}-E\left(y_{i,\text{rep}}|Y_{\mathrm{vec}}\right)\right)}^{2}+\sum_{i=1}^{2MJK}V\text{ar}\left(y_{i,\text{rep}}|Y_{\mathrm{vec}}\right),$$

where $Y_{\mathrm{vec}}={\left(y_{1,\text{vec}},y_{2,\text{vec}},\ldots ,y_{2MJK,\text{vec}}\right)}^{\prime }$ is a vector of length *2MJK* obtained by vectorizing observed data array **Y** and **Y**_rep_ = (y_1,rep_, y_2,rep_,…,y_2MJK_,_rep_)′ represents the vector of all these replicates. The first term in the *D*_∞_ criterion (see Equation 13) is the goodness‐of‐fit term, while the second term can be interpreted as a penalty term for model complexity. The model with the smallest *D*_∞_ is to be preferred. In our analysis, the two data sets are obtained by vectorizing the two binary data arrays.

We compute the above expectation $E\left(y_{i,\text{rep}}|Y_{\text{vec}}\right)$ and variance $V\text{ar}\left(y_{i,\text{rep}}|Y_{\text{vec}}\right)$ using the MCMC draws. Given an MCMC sample **µ** ^(d)^: *d* = 1,…,*N*_iter_ from π(*µ*|**Y**), we simulate $Y_{\mathrm{rep}}^{\left(d\right)}$ from *f*(**Y**|**µ** ^(^ *^d^* ^)^) for each *d* = 1,…,*N*_iter_. For instance, in model *M*_1_, *µ* denotes the collection of the parameters *ψ*, *θ*, *ϕ*, *ω*_0_, *σ_m_*, σ_f_, **u**_0_, **z**, **S**, **L**. Then $E\left(y_{i,\text{rep}}|Y_{\text{vec}}\right)\approx N_{\text{iter}}^{-1}\sum_{d=1}^{N_{\text{iter}}}y_{i,\text{rep}}^{\left(d\right)}$ and $\text{Var}\left(y_{i,\text{rep}}|Y_{\text{vec}}\right)\approx N_{\text{iter}}^{-1}$ $\sum_{i=1}^{N_{\text{iter}}}{\left(y_{i,\text{rep}}^{\left(d\right)}-N_{\text{iter}}^{-1}\sum_{d=1}^{N_{\text{iter}}}y_{i,\text{rep}}^{\left(d\right)}\right)}^{2}$. We summarize the various model selection methods and their variants in Table 5. Considering all these model selection tools and their variants (from approximation approaches to setting tuning densities), our evaluation is carried out on 25 unique tools. By no means is this an exhaustive list of candidate tools because there are a large number of tools available. Our purpose here really is to assess some popular tools and assess their efficacies on a particular class of models.

**Table 5:** Bayesian model selection methods used in this study

Sl. no.	Model selection method	Variant	Approximation method	Choices of tuning density	Eq. No.
1.	Bayes factor	Gelfand‐Dey estimator	MAP	Multivariate normal density, multivar‐ iate‐*t* density with degrees of freedom 10, 100, 500, 1,000, 10,000 and truncated multivariate normal density with confidence coefficients 0.90, 0,95, 0.99	(7)
2.	Bayes factor	Gelfand‐Dey estimator	IL	‐Do‐	(8)
3.	Bayes factor	Harmonic mean estimator	—	—	(6)
4.	DIC	*p*_DIC1_	MAP	—	(10)
5.	DIC	*p*_DIC2_	MAP	—	(10)
6.	WAIC	*p*_WAIC1_	—	—	(11)
7.	WAIC	*p*_WAIC2_	—	—	(11)
8.	WAIC	*p*_WAIC3_	—	—	(12)
9.	Posterior predictive loss	—	—	—	(13)

## 3 EVALUATION OF THE PERFORMANCE OF MODEL SELECTION METHODS

### 3.1 Simulation design and simulation scenarios

**Figure 1:**
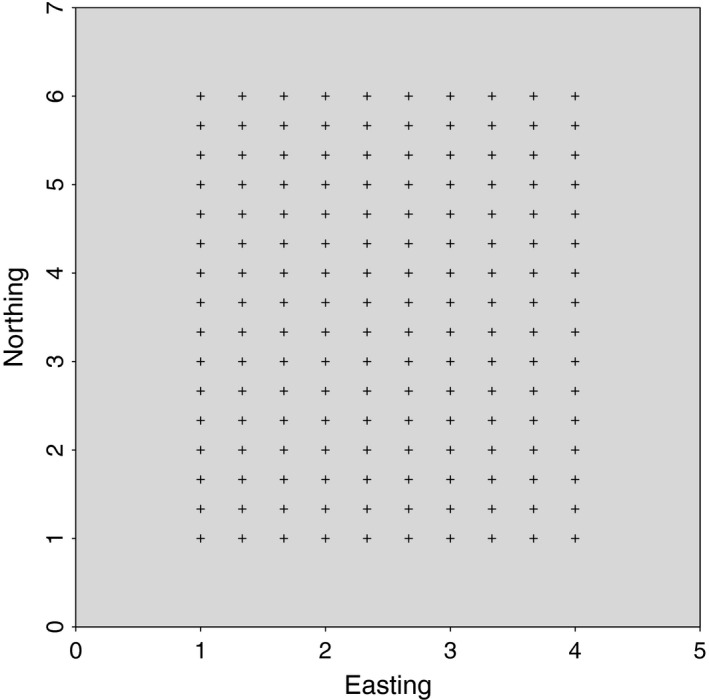
Array of trap locations (denoted by “+”) within the state space (0.5) × (0.7)

We have conducted simulations for 12 scenarios (provided in Table 6) grouped into 2 equal sized sets, to assess the performance of the models proposed here. We set *σ_m_* = 0.3 and *σ_f_* = 0.15 for the first set of 6 scenarios, *σ_m_* = 0.4 and *σ_f_* = 0.2 for the second set of 6 scenarios. We set (*ω*_0_, *ϕ*) = {(0.01, 0.3), (0.05, 0.3), (0.05, 0.5), (0.03, 0.8), (0.01, 0.9), (0.05, 0.9)}, which gives us 6 different scenarios for each of the two sets corresponding to the values taken by *ω*_0_ and *ϕ*. We assume that a total of 100 individuals are residing inside the state space of which 40 are male. Each of the simulation experiments is conducted within a rectangular state space of dimension 5 unit × 7 unit (Figure 1), after setting a buffer of 1 unit in both horizontal and vertical directions, a 10 × 16 trapping array of total *J* = 160 trap stations has been set (trap spacing is 0.3 unit on *X* axis and 0.3125 unit on *Y* axis). This meets the requirement suggested in Karanth and Nichols (2017). Each of the traps remains active for *K* = 50 sampling occasions simultaneously. For parameter estimation, we set the maximum possible number of individuals present in the population (*M*) at 400 for all the scenarios. Since MCMC approaches are computationally time consuming, we set the data‐generating parameters to generate data sets of varying levels of information content. This strategy ensured that we capture trends in model selection performance by the different methods. The experiment is repeated *n*_sim_ = 10 times. The MCMC samples for each of the parameters are obtained (each of length 30,000), and the estimates are computed using those chains after a burn‐in of 10,000. It takes approximately 3 days to run a single chain of 30,000 iterations using our R code for model *M*_1_, approximately 2 days for model *M*_2_ and *M*_3_, and approximately 1 day for model *M*_4_ using our R code on a DELL Precision Rack 7,910 Server with 40 cores and 512 GB RAM at a clock speed of 2.20 GHz.

**Table 6:** Parameter specifications corresponding to different simulation scenarios

Scenario	*M*	*N*	*N*_Male_	*ω*_0_	*ϕ*	*σ_m_*	*σ_f_*
1	400	100	40	0.01	0.3	0.3	0.15
2	400	100	40	0.01	0.9	0.3	0.15
3	400	100	40	0.01	0.3	0.4	0.20
4	400	100	40	0.01	0.9	0.4	0.20
5	400	100	40	0.03	0.8	0.3	0.15
6	400	100	40	0.03	0.8	0.4	0.20
7	400	100	40	0.05	0.3	0.3	0.15
8	400	100	40	0.05	0.5	0.3	0.15
9	400	100	40	0.05	0.9	0.3	0.15
10	400	100	40	0.05	0.3	0.4	0.20
11	400	100	40	0.05	0.5	0.4	0.20
12	400	100	40	0.05	0.9	0.4	0.20

Capture–recapture data sets are simulated independently under each of the 12 simulation scenarios (Table 6) under model *M*_1_. Recall that, model *M*_1_ corresponds to the statistical model in (Equation 1) with *σ* parameter modeled in terms of individual covariate on sex category **u** (see Section 2.1). Then, each simulated data set is fitted with all the four competing model *M*_1_, *M*_2_, *M*_3_, and *M*_4_. For a better mixing during the MCMC run, we transform the components of ***µ*** *_p_* so that the transformed parameter space is the entire Euclidean space (details are given in Appendix S1C).

### 3.2 Defining performance measures

#### 3.2.1 Probability of selecting the true model

In our study, since all the data sets are simulated from model *M*_1_, it is considered as the true model. We have computed the proportion of times a model selection method chooses *M*_1_ as the best out of *n* = 10 simulations. This proportion will serve as an estimate for the probability of selecting the true model. Additionally, we have also computed the proportion of times a model selection method chooses *M*_2_, *M*_3_, and *M*_4_, respectively. The computed proportions of selecting these models from the simulation study will indicate the efficacy of the model selection methods.

#### 3.2.2 Assessing the quality of parameter estimation

The abundance parameter *N* carries a lot of significance in ecology and conservation. Due to its importance, ecologists place their interests in the robustness and accuracy of its estimate, and will therefore rely on a model selection method that will achieve this by selecting that model from the model set that will provide the most reliable estimate of *N*. The precision and accuracy of the parameter estimate indicates the quality of the model fit, and we assess this by computing the *average root mean square error* (average RMSE).

Suppose {*µ* ^(^ *^td^* ^)^: *d* = 1, …, *N*_iter_} denotes a set of MCMC draws from the posterior distribution of an arbitrary parameter *µ* for the *t*‐th simulated data set, *t* = 1,…,*n*_sim_. *Mean square error* (*MSE*) of *µ* for the *t*‐th simulated data set is estimated as $\text{MSE}\left(\mu ,\;t\right)=N_{\text{iter}}^{-1}\sum_{d=1}^{N_{\text{iter}}}{\left(\mu^{\left(td\right)}-\mu \right)}^{2}.$ Average RMSE is calculated by first averaging the estimated MSEs of different simulations and then taking the square root of the average:

$$\text{Average}\;\text{RMSE}\left(\mu \right)=\sqrt{n_{\text{sim}}^{-1}\sum_{t=1}^{n_{\text{sim}}}\text{MSE}(\mu ,\;t)}.$$

Let us now summarize our experiment. The quantities related to the various Bayesian model selection methods (Section 2.2) are computed for each of the four models for the simulated data and analysis sets. We compare the conclusions drawn from the computed proportions with the conclusions drawn from average RMSEs of the parameters to study the behavior of the model selection methods with respect to varying information content. We also generate pairwise correlation plots from the MCMC draws to study the extent of parameter redundancy between various pairs of parameters as a consequence of lack of information content in the data.

## 4 RESULTS OF THE SIMULATION STUDY

**Figure 2:**
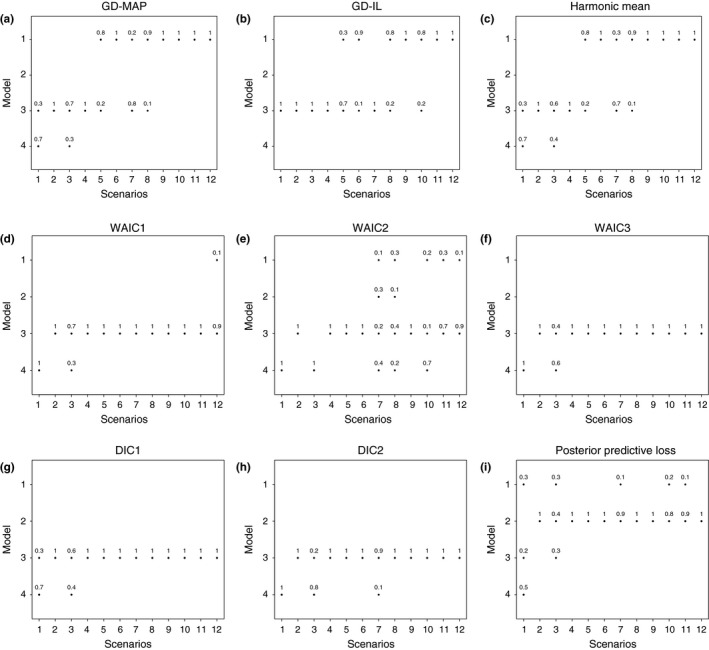
Plot (a): The proportion of times Gelfand‐Dey estimator of Bayes factor favors any particular model using the MAP approximation approach and a multivariate normal density for *g*. Plot (b): The proportion of times Gelfand‐Dey estimator of Bayes factor favors any particular model using the integrated likelihood approximation approach and a multivariate normal density for *g*. Plot (c): The proportion of times harmonic mean estimator of Bayes factor using the favors any particular model. Plots (d)–(i) correspond to WAIC1, WAIC2, WAIC3, DIC1, DIC2, and posterior predictive loss, respectively

Figure 2 shows the proportion of times different estimators of Bayes factor favors any particular model. The subplots have different scenarios 1–12 in the *x*‐axis and the competing models *M*_1_–*M*_4_ in the *y*‐axis. Plot (a) and Plot (b) correspond to the Gelfand‐Dey estimator of the Bayes factor favoring any particular model using (i) the MAP approximation approach and a multivariate normal density for tuning density *g*, (ii) the integrated likelihood approximation approach and a multivariate normal density for tuning density *g*, respectively. Plot (c) corresponds to the harmonic mean estimator of the Bayes factor. Plots (d–i) correspond to WAIC1, WAIC2, WAIC3, DIC1, DIC2, and posterior predictive loss, respectively.

Our results suggest that the choice of the tuning density in computation of Gelfand‐Dey estimator had no impact on model selection (Figure 2). Thus, we focus only on the results corresponding to the normal tuning density. We observe that the Bayes factor with the GD‐MAP approximation favors *M*_4_ in 70% of the times under scenario 1 and favors *M*_3_ in at least 70% of the times under scenarios 2, 3, 4, and 7. Bayes factor (GD‐MAP) is in favor of *M*_1_ more than 80% of the times under all the remaining scenarios 5–6 and 8–12. As seen here, the GD‐IL approximation performs slightly worse than the GD‐MAP approximation (Figure 2). However, surprisingly, we observe that the model choices by the harmonic mean estimator of Bayes factor performs well and favors the true model *M*_1_ in majority of the scenarios.

We have considered three forms of the WAIC and two forms of the DIC. The corresponding plots are shown in Figure 2(d–h). WAIC1, WAIC3, DIC1, and DIC2 exhibit very similar tendencies in their model choices by favoring *M*_3_ under all the scenarios except scenarios 1 and 3. These four methods favor *M*_4_ under scenario 1. Under scenario 3, WAIC1 and DIC1 favor *M*_3_ in majority of the times, whereas WAIC3 and DIC2 favor *M*_4_. Like Bayes factor, these model selection methods (DICs and WAICs) also tend to favor simpler models under scenarios 1 and 3. The WAIC2 largely agrees with the other WAICs (and DICs) but more often selects the true model when data sets are more informative (Figure 2d–f). In general, DICs and WAICs seem to discourage the presence of high‐dimensional latent variables.

The plot showing the proportion of different model choices by posterior predictive loss is given in Figure 2i. The posterior predictive loss criterion *D*_∞_ favors models with individual sex covariates (*M*_1_ and *M*_2_). The posterior predictive loss also appears to select the true model some of the times, even when there is not sufficient information in the data (scenarios 1 and 3).

**Figure 3:**
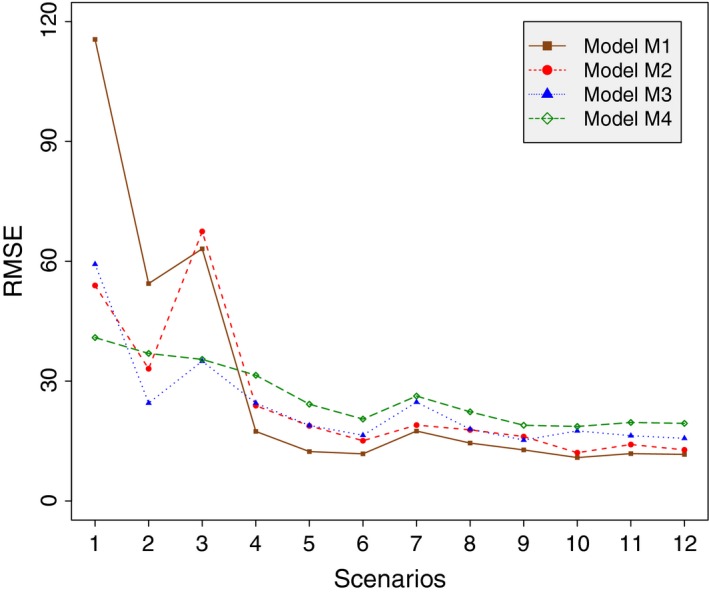
Plot of average RMSE estimates of *N* over different simulation scenarios

In Figure 3, we see that the average RMSE of *N* is substantially higher in scenarios 1–3 relative to scenarios 4–12. We also have observed a substantial amount of correlation between some pairs of parameters involving *N* in the MCMC chains under scenarios 1–3 (*r*(*N*, *θ*) ≈ −0.7, *r*(*N*, *σ_f_* ≈ −0.42). However, these correlations decrease for the other scenarios, likely due to increased information content in the data. For interested readers, we have also provided additional plots for the model selection methods (Figures 1–6), the RMSE plots for all the parameters (Figures 7 and 8), and a representative set of scatter plots (Figures 9–14) in the Appendix S1E.

## 5 DISCUSSION AND CONCLUSIONS

Contemporary practice of ecology and conservation biology relies largely on the use of model selection for hypotheses discrimination (Johnson & Omland, 2004). Simultaneously, there has been major growth in the use of hierarchical models in ecology, especially within the realm of Bayesian inference (Kéry & Royle, 2015; Royle & Dorazio, 2008). These models have now enabled statistical ecologists to fairly easily formulate complex ecological models, elegantly deal with the sampling process and also fit these complex models using powerful tools such as the MCMC (Kéry & Royle, 2015). However, the lack of availability of ready‐made model selection tools when practicing the Bayesian inference has sometimes motivated ecologists to continue using the likelihood‐based inferences, merely because one can use well‐known model selection tools such as the AIC (Burnham & Anderson, 2002) for inference.

To provide a context for this argument, in the spatial capture–recapture literature, we have essentially seen the development of three important likelihood functions: (1) Borchers and Efford (2008), (2) Royle et al. (2009) and (3) Royle, Sutherland, Fuller, and Sun (2015). Inferences for the models (1) and (3) are by maximizing the likelihood, while the inference for (2) is Bayesian. We note with interest that one of the reasons for the development of (3) was motivated on the pretext that model selection is much easier (using known tools such as the AIC) for practitioners using the likelihood approach, in spite of the problem having been already solved in the Bayesian context (Royle et al., 2013). It is specially of concern in the context of the models we study here in that investigators may be forced to integrate out **S** (activity centers of individuals) in order to construct tractable likelihoods and thus oversimplifying ecological reality. For example, by retaining the activity centers it is possible for investigators to confront open SCR models (Gardner, Sollmann, Kumar, Jathanna, & Karanth, 2018). We therefore hope that our study motivates the continued use of Bayesian methods by investigators for all its advantages, instead of opting out of them merely for the sake of using simple model selection methods.

In this study, we have tried to implement some selected Bayesian model selection methods on a specific class of advanced Bayesian SCR models (Royle, 2015—with and without sex covariates; Dey et al. (2019)–with and without sex covariates) dealing with partially identified individuals. We have found our Bayes factor implementation using the Gelfand‐Dey estimator (using the MAP approximation approach) to be the preferred choice as a model selection method over a wide range of simulation scenarios. This approach appears to work particularly well when information content in the data is moderate to high. The IL approximation approach also worked well but not as well as the MAP approximation approach perhaps because there exists interdependency between some of the latent variables which are not accurately captured during marginalization.

However, implementing Bayes factors for model selection using the Gelfand‐Dey estimator (with MAP approximation) is not very straightforward and requires investigators to incorporate some code during model fitting by MCMC. Interestingly, our study demonstrates that obtaining Bayes factors using the harmonic mean approach for marginal likelihood computation is less demanding but yet serves as a very good model selection method. This finding deviates from the popular view among applied scientists that it is futile to estimate the marginal likelihood using the harmonic mean approach (Lartillot & Philippe, 2006; Xie, Lewis, Fan, Kuo, & Chen, 2011). We surmise that this finding may be attributed to the fact that when the priors are bounded (as we have done, but for other reasons) and does not permit extremely low probabilities to occur at the tails. Further, our emphasis here is to ask how the tool performs to select the true model and estimate parameters for prediction for a given analytical setup. And it is not about how accurately the tools estimate the actual value of the marginal likelihood. Thus, many of the criticisms (Lartillot & Philippe, 2006; Xie et al., 2011) may become irrelevant in practice, but this would require further enquiry.

As our simulation study shows, the two goals of model selection and parameter estimation cannot be simultaneously achieved under certain circumstances (scenarios 1–3), especially when information content is low (indicated by high RMSE values and correlation coefficients). Hence, researchers have to clearly prioritize their objectives prior to data analysis. If the goal, for example, is to find a model that best estimates population size *N*, then we recommend the use of Bayes factor (Gelfand‐Dey with MAP approximation) or the Bayes factor (harmonic mean, due to its simplicity) because these appear to provide the most reliable estimates of *N* over all the simulation scenarios. However, if researchers are only interested to select the true model, especially when data are less informative (scenarios 1–3), we recommend the posterior predictive loss approach since they favor the true model nearly a one‐third of the times only in situations with such low information content. Of course, the dual objectives of model selection and parameter estimation are met when information content is moderate or high (scenarios 4–12), and as stated previously, we recommend either the Bayes factor (Gelfand‐Dey with MAP approximation) or the Bayes factor (harmonic mean) in such cases. However, the posterior predictive loss (with the squared error loss function) as used here does not select the true model when information content is moderate to high.

We also do not recommend the use of the DICs or the WAICs, since they do not appear to outcompete other model selection tools, either from the standpoint of model selection or parameter estimation, in any of the simulation scenarios. This is an interesting finding, because tools such as the WAIC are much newer tools developed by applied Bayesians and recommended for hierarchical models (Gelman, Carlin, et al., 2014; Hooten & Hobbs, 2015). Thus, our study brings back focus on the need to assess the strength of inference from a model selection method by *conditioning* on a true model and consequently evaluating a *competing* set of model selection methods prior to selecting the most appropriate one for the problem on hand. Table 7 summarizes the similarities and differences in inferences between our specific evaluation of model selection tools and the generally accepted evaluations. We believe this summary will motivate investigators to look deeper into their own models and the model selection methods they are using.

**Table 7:** Contrasting the performance of model selection tools based on the intended purpose and perceived applicability with findings from our specific study. In the table, we provide answers to the following questions: (a) Does this approach select the true model? (b) Does this approach favor models providing reliable estimates of parameters (specifically for *N*)? (c) How difficult is the approach to implement in practice? Comments in bold draw attention to the noticeable differences between the expected performance of a tool and its performance in our particular study

Model selection tool	Intended purpose and applicability	Reference	Findings from our specific study
Bayes factor (by Gelfand‐Dey estimator)	(a) Yes (b) Yes (c) Difficult		(a) Yes, very often (MAP) Yes, quite often (IL) (b) Yes, very often (MAP) Yes, quite often (IL) (c) Moderately difficult (MAP) Moderately difficult (IL)
Bayes factor (by harmonic mean estimator)	(a) **Yes, but unreliable** (b) Yes (c) Easy	Newton and Raftery (1994), and Kass and Raftery (1995)	(a) **Yes, very often** (b) Yes, very often (c) Easy
DIC1	(a) No (b) **Yes** (c) **Easy**	Spiegelhalter et al. (2002), Gelman, Hwang, & Vehtari, 2014), and Hooten and Hobbs (2015)	(a) No (b) **No** (c) **Moderately difficult**
DIC2	(a) No (b) **Yes** (c) **Easy**	Spiegelhalter et al. (2002), Gelman, Hwang, & Vehtari, 2014), and Hooten and Hobbs (2015)	(a) No (b) **No** (c) **Moderately difficult (required MAP estimate)**
WAIC1	(a) No (b) **Yes** (c) Easy	Watanabe (2010), Gelman, Hwang, & Vehtari, 2014), and Hooten and Hobbs (2015)	(a) No (b) **No** (c) Easy
WAIC2	(a) **No** (b) **Yes** (c) Easy	Watanabe (2010), Gelman, Hwang, & Vehtari, 2014), Hooten and Hobbs (2015)	(a) **No, not often** (b) **Yes, but suboptimally** (c) Easy
WAIC3	(a) No (b) **Yes** (c) Easy		(a) No (b) **No** (c) Easy
Posterior predictive loss	(a) **No** (b) **Yes** (c) Moderately difficult	Gelfand and Ghosh (1998), and Hooten and Hobbs (2015)	(a) **No, not often** (b) **Yes, but suboptimally** (c) Moderately difficult

Our approach does not, strictly speaking, permit us to draw conclusions and make inferences on the most suitable model selection tools beyond the restrictive set of competing models and the settings we have used in this study. However, we demonstrate the applicability of the Bayes factor on a set of structurally very complex hierarchical models. This was possible by using our approach of partitioning the real‐valued scalar and high‐dimensional structural parameters. We anticipate that this general approach could address difficulties faced by ecologists attempting to use the Bayes factors for comparing very complex hierarchical models. We would expect that it would be much easier to use the Bayes factor for relatively simpler and a much larger class of hierarchical models (Kéry & Royle, 2015; Royle et al., 2013). We note with interest that it is also unclear whether the routinely used AIC works as an appropriate model selection tool for MLE‐based SCR models as discussed in Efford and Mowat (2014). In real‐world problems, we can almost always find proper priors for parameters to ensure that Bayes factors exist. Therefore, we believe it is best to depend on tools built on sound statistical theory (by finding suitable approximations) rather than seeking answers based on seemingly elegant but not well‐tested tools.

## CONFLICT OF INTEREST

None declared.

## AUTHORS CONTRIBUTION

SD, MD, and AMG conceived the ideas and designed methodology; SD conducted data analysis; SD, MD, and AMG drafted the article. All authors contributed critically to the drafts and gave final approval for publication.

## Data Availability

R codes are available from GitHub, https://github.com/soumenstat89/bmse.git. Authors have used only simulated data sets in this article, which can be generated using the R codes provided. Hence, no particular data set is archived.
