# Supplementary material for: Bayesian model selection for spatial capture–recapture models
Source: Ecol Evol. 2019 Sep 30;9(20):11569–83. doi: 10.1002/ece3.5551 (PMC6822056; doi:10.1002/ece3.5551)
Supplement: Supplementary file 1 [file ECE3-9-11569-s001.pdf]

Appendix

Bayesian Model Selection for Spatial Capture Recapture Models

Soumen Dey[1], Mohan Delampady[1], and Arjun M. Gopalaswamy *[1]

[1]Statistics and Mathematics Unit, Indian Statistical Institute, Bangalore 560059, India

A Steps of resampling procedure to obtain MAP estimate using MCMC draws

We describe a MAP approximation approach to compute the Gelfand-Dey estimator of the Bayes factor in Section 2.2.1 of the main text. We denote the collection of all parameters $\boldsymbol{\mu} = (\boldsymbol{\mu_p}, \boldsymbol{\mu_s})$, where $\boldsymbol{\mu_p}$ is the collection of scalar parameters and $\boldsymbol{\mu_s}$ is the collection of all latent variables. We first fix these high-dimensional variables at their MAP estimate $\hat{\boldsymbol{\mu}}_{\boldsymbol{s}}$ assuming that the posterior distribution is well summarised by this estimate. The Gelfand-Dey estimator of marginal likelihood of data $m(\mathbf{Y})$ is then computed as (also given as (2.7) in the main text):

$$\hat{m}_{\mathrm{GD}}(\mathbf{Y}) = \left[\frac{1}{N_{iter}} \sum_{d=1}^{N_{iter}} \frac{g({\boldsymbol{\mu_p}}^{(d)})}{f(\mathbf{Y} \,|\, {\boldsymbol{\mu_p}}^{(d)}, \hat{\boldsymbol{\mu}}_{\boldsymbol{s}})\, \pi({\boldsymbol{\mu_p}}^{(d)})} \right]^{-1}. \tag{A.1}$$

where $\{({\boldsymbol{\mu_p}}^{(d)}, {\boldsymbol{\mu_s}}^{(d)}) \,:\, d = 1, \ldots, N_{iter}\}$ is a set of MCMC draws from the posterior $\pi(\boldsymbol{\mu_p}, \boldsymbol{\mu_s} \,|\, \mathbf{Y})$, $f(\mathbf{Y} \,|\, \boldsymbol{\mu_p}, \boldsymbol{\mu_s})$ denotes the model likelihood and $\pi(\boldsymbol{\mu_p})$ denotes the prior density of the parameters $\boldsymbol{\mu_p}$ and $g(\boldsymbol{\mu_p})$ denotes a tuning density. We begin with

* *arjungswamy@gmail.com* (✉)

$(\boldsymbol{\mu_p}^{(d_0)}, \boldsymbol{\mu_s}^{(d_0)})$ as an initial estimate of $(\boldsymbol{\mu_p}, \boldsymbol{\mu_s})$ where

$$f(\mathbf{Y} \mid \boldsymbol{\mu_p}^{(d_0)}, \boldsymbol{\mu_s}^{(d_0)})\, \pi(\boldsymbol{\mu_p}^{(d_0)}, \boldsymbol{\mu_s}^{(d_0)}) = \max_d \{f(\mathbf{Y} \mid \boldsymbol{\mu_p}^{(d)}, \boldsymbol{\mu_s}^{(d)})\, \pi(\boldsymbol{\mu_p}^{(d)}, \boldsymbol{\mu_s}^{(d)})\}.$$

This estimate of posterior mode of $(\boldsymbol{\mu_p}, \boldsymbol{\mu_s})$ may not be optimal since in our high dimensional parameter setting, an MCMC sample of a practical size may not be enough to extensively explore the posterior surface. Below we describe a resampling algorithm based on MCMC draws to obtain a better MAP estimate $(\hat{\boldsymbol{\mu}}_{\boldsymbol{p}}, \hat{\boldsymbol{\mu}}_{\boldsymbol{s}})$.

Iteration 1. Obtain d_1 such that

$$f(\mathbf{Y} \mid \boldsymbol{\mu_p}^{(d_1)}, \boldsymbol{\mu_s}^{(d_0)})\, \pi(\boldsymbol{\mu_p}^{(d_1)}, \boldsymbol{\mu_s}^{(d_0)}) = \max_d \{f(\mathbf{Y} \mid \boldsymbol{\mu_p}^{(d)}, \boldsymbol{\mu_s}^{(d_0)})\, \pi(\boldsymbol{\mu_p}^{(d)}, \boldsymbol{\mu_s}^{(d_0)})\}.$$

- If $f(\mathbf{Y} \mid \boldsymbol{\mu_p}^{(d_1)}, \boldsymbol{\mu_s}^{(d_0)})\, \pi(\boldsymbol{\mu_p}^{(d_1)}, \boldsymbol{\mu_s}^{(d_0)}) \le f(\mathbf{Y} \mid \boldsymbol{\mu_p}^{(d_0)}, \boldsymbol{\mu_s}^{(d_0)})\, \pi(\boldsymbol{\mu_p}^{(d_0)}, \boldsymbol{\mu_s}^{(d_0)})$

  $\longrightarrow$ Stop the iterations, set $(\hat{\boldsymbol{\mu}}_{\boldsymbol{p}}, \hat{\boldsymbol{\mu}}_{\boldsymbol{s}}) = (\boldsymbol{\mu_p}^{(d_0)}, \boldsymbol{\mu_s}^{(d_0)})$.
- Otherwise go to the next iteration.

Iteration 2. Obtain d_2 such that

$$f(\mathbf{Y} \mid \boldsymbol{\mu_p}^{(d_1)}, \boldsymbol{\mu_s}^{(d_2)})\, \pi(\boldsymbol{\mu_p}^{(d_1)}, \boldsymbol{\mu_s}^{(d_2)}) = \max_d \{f(\mathbf{Y} \mid \boldsymbol{\mu_p}^{(d_1)}, \boldsymbol{\mu_s}^{(d)})\, \pi(\boldsymbol{\mu_p}^{(d_1)}, \boldsymbol{\mu_s}^{(d)})\}.$$

- If $f(\mathbf{Y} \mid \boldsymbol{\mu_p}^{(d_1)}, \boldsymbol{\mu_s}^{(d_2)})\, \pi(\boldsymbol{\mu_p}^{(d_1)}, \boldsymbol{\mu_s}^{(d_2)}) \le f(\mathbf{Y} \mid \boldsymbol{\mu_p}^{(d_1)}, \boldsymbol{\mu_s}^{(d_0)})\, \pi(\boldsymbol{\mu_p}^{(d_1)}, \boldsymbol{\mu_s}^{(d_0)})$

  $\longrightarrow$ Stop the iterations, set $(\hat{\boldsymbol{\mu}}_{\boldsymbol{p}}, \hat{\boldsymbol{\mu}}_{\boldsymbol{s}}) = (\boldsymbol{\mu_p}^{(d_1)}, \boldsymbol{\mu_s}^{(d_0)})$.
- Otherwise go to the next iteration.

Iteration 3. Obtain d_3 such that

$$f(\mathbf{Y} \mid \boldsymbol{\mu_p}^{(d_3)}, \boldsymbol{\mu_s}^{(d_2)})\, \pi(\boldsymbol{\mu_p}^{(d_3)}, \boldsymbol{\mu_s}^{(d_2)}) = \max_d \{f(\mathbf{Y} \mid \boldsymbol{\mu_p}^{(d)}, \boldsymbol{\mu_s}^{(d_2)})\, \pi(\boldsymbol{\mu_p}^{(d)}, \boldsymbol{\mu_s}^{(d_2)})\}.$$

- If $f(\mathbf{Y} \mid \boldsymbol{\mu_p}^{(d_3)}, \boldsymbol{\mu_s}^{(d_2)})\, \pi(\boldsymbol{\mu_p}^{(d_3)}, \boldsymbol{\mu_s}^{(d_2)}) \le f(\mathbf{Y} \mid \boldsymbol{\mu_p}^{(d_1)}, \boldsymbol{\mu_s}^{(d_2)})\, \pi(\boldsymbol{\mu_p}^{(d_1)}, \boldsymbol{\mu_s}^{(d_2)})$

  $\longrightarrow$ Stop the iterations, set $(\hat{\boldsymbol{\mu}}_{\boldsymbol{p}}, \hat{\boldsymbol{\mu}}_{\boldsymbol{s}}) = (\boldsymbol{\mu_p}^{(d_1)}, \boldsymbol{\mu_s}^{(d_2)})$.
- Otherwise go to the next iteration.

Iteration 4. Obtain d_4 such that

$$f(\mathbf{Y} \mid {\boldsymbol{\mu_p}}^{(d_3)}, {\boldsymbol{\mu_s}}^{(d_4)})\, \pi({\boldsymbol{\mu_p}}^{(d_3)}, {\boldsymbol{\mu_s}}^{(d_4)}) = \max_d \{ f(\mathbf{Y} \mid {\boldsymbol{\mu_p}}^{(d_3)}, {\boldsymbol{\mu_s}}^{(d)})\, \pi({\boldsymbol{\mu_p}}^{(d_3)}, {\boldsymbol{\mu_s}}^{(d)}) \}.$$

- If $\quad f(\mathbf{Y} \mid {\boldsymbol{\mu_p}}^{(d_3)}, {\boldsymbol{\mu_s}}^{(d_4)})\, \pi({\boldsymbol{\mu_p}}^{(d_3)}, {\boldsymbol{\mu_s}}^{(d_4)}) \leq f(\mathbf{Y} \mid {\boldsymbol{\mu_p}}^{(d_3)}, {\boldsymbol{\mu_s}}^{(d_2)})\, \pi({\boldsymbol{\mu_p}}^{(d_3)}, {\boldsymbol{\mu_s}}^{(d_2)})$

  $\longrightarrow$ Stop the iterations, set $(\hat{\boldsymbol{\mu}}_{\boldsymbol{p}}, \hat{\boldsymbol{\mu}}_{\boldsymbol{s}}) = ({\boldsymbol{\mu_p}}^{(d_3)}, {\boldsymbol{\mu_s}}^{(d_2)})$.

- Otherwise go to the next iteration.

$\vdots$

$\vdots$

Iteration m. (i) If m is even, obtain d_m such that

$$f(\mathbf{Y} \mid {\boldsymbol{\mu_p}}^{(d_{m-1})}, {\boldsymbol{\mu_s}}^{(d_m)})\, \pi({\boldsymbol{\mu_p}}^{(d_{m-1})}, {\boldsymbol{\mu_s}}^{(d_m)}) = \max_d \{ f(\mathbf{Y} \mid {\boldsymbol{\mu_p}}^{(d_{m-1})}, {\boldsymbol{\mu_s}}^{(d)})\, \pi({\boldsymbol{\mu_p}}^{(d_{m-1})}, {\boldsymbol{\mu_s}}^{(d)}) \}.$$

- If $\quad f(\mathbf{Y} \mid {\boldsymbol{\mu_p}}^{(d_{m-1})}, {\boldsymbol{\mu_s}}^{(d_m)})\, \pi({\boldsymbol{\mu_p}}^{(d_{m-1})}, {\boldsymbol{\mu_s}}^{(d_m)})$
  $\leq f(\mathbf{Y} \mid {\boldsymbol{\mu_p}}^{(d_{m-1})}, {\boldsymbol{\mu_s}}^{(d_{m-2})})\, \pi({\boldsymbol{\mu_p}}^{(d_{m-1})}, {\boldsymbol{\mu_s}}^{(d_{m-2})})$

  $\longrightarrow$ Stop the iterations, set $(\hat{\boldsymbol{\mu}}_{\boldsymbol{p}}, \hat{\boldsymbol{\mu}}_{\boldsymbol{s}}) = ({\boldsymbol{\mu_p}}^{(d_{m-1})}, {\boldsymbol{\mu_s}}^{(d_{m-2})})$.

- Otherwise go to the next iteration.

(ii) If m is odd, obtain d_m such that

$$f(\mathbf{Y} \mid {\boldsymbol{\mu_p}}^{(d_m)}, {\boldsymbol{\mu_s}}^{(d_{m-1})})\, \pi({\boldsymbol{\mu_p}}^{(d_m)}, {\boldsymbol{\mu_s}}^{(d_{m-1})}) = \max_d \{ f(\mathbf{Y} \mid {\boldsymbol{\mu_p}}^{(d)}, {\boldsymbol{\mu_s}}^{(d_{m-1})})\, \pi({\boldsymbol{\mu_p}}^{(d)}, {\boldsymbol{\mu_s}}^{(d_{m-1})}) \}.$$

- If $\quad f(\mathbf{Y} \mid {\boldsymbol{\mu_p}}^{(d_m)}, {\boldsymbol{\mu_s}}^{(d_{m-1})})\, \pi({\boldsymbol{\mu_p}}^{(d_m)}, {\boldsymbol{\mu_s}}^{(d_{m-1})})$
  $\leq f(\mathbf{Y} \mid {\boldsymbol{\mu_p}}^{(d_{m-2})}, {\boldsymbol{\mu_s}}^{(d_{m-1})})\, \pi({\boldsymbol{\mu_p}}^{(d_{m-2})}, {\boldsymbol{\mu_s}}^{(d_{m-1})})$

  $\longrightarrow$ Stop the iterations, set $(\hat{\boldsymbol{\mu}}_{\boldsymbol{p}}, \hat{\boldsymbol{\mu}}_{\boldsymbol{s}}) = ({\boldsymbol{\mu_p}}^{(d_{m-2})}, {\boldsymbol{\mu_s}}^{(d_{m-1})})$.

- Otherwise go to the next iteration.

This procedure is continued iteratively to eventually give us the best MAP estimate of the posterior mode $(\hat{\boldsymbol{\mu}}_{\boldsymbol{p}}, \hat{\boldsymbol{\mu}}_{\boldsymbol{s}})$. Observe that, $(\hat{\boldsymbol{\mu}}_{\boldsymbol{p}}, \hat{\boldsymbol{\mu}}_{\boldsymbol{s}})$ is closer to the posterior mode than $({\boldsymbol{\mu_p}}^{(d_0)},\, {\boldsymbol{\mu_s}}^{(d_0)})$ since

$$f(\mathbf{Y} \mid \hat{\boldsymbol{\mu}}_{\boldsymbol{p}}, \hat{\boldsymbol{\mu}}_{\boldsymbol{s}}))\, \pi(\hat{\boldsymbol{\mu}}_{\boldsymbol{p}}, \hat{\boldsymbol{\mu}}_{\boldsymbol{s}}) \geq f(\mathbf{Y} \mid {\boldsymbol{\mu_p}}^{(d_0)}, {\boldsymbol{\mu_s}}^{(d_0)})\, \pi({\boldsymbol{\mu_p}}^{(d_0)}, {\boldsymbol{\mu_s}}^{(d_0)}).$$

Thus the asymptotic (large posterior sample) convergence of $(\boldsymbol{\mu_p}^{(d_0)}, \boldsymbol{\mu_s}^{(d_0)})$ to the posterior mode ensures that our improved MAP estimate also converges to the same point. Note that we have ensured via suitable transformations (see Appendix C) on the parameters that all the points in $\{(\boldsymbol{\mu_p}^{(a)}, \boldsymbol{\mu_s}^{(b)}) \, : \, a, b = 1, \ldots, N_{iter}; a \neq b\}$ do belong to the posterior support. Indeed, we have simply used the MCMC sample itself to explore the posterior surface better, and thus have obtained an improved MAP estimate.

B Integrated likelihoods

B.1 Integrated likelihood under M_1

The joint density of $\mathbf{Y}^* := (\mathbf{Y^{(1)}}, \mathbf{Y^{(2*)}}) = ((y_{ijk}^{(1)}, y_{ijk}^{(2*)}))$ and $\mathbf{u} = (u_1, \ldots, u_M)'$ under M_1 is the following (also given as model likelihood (2.1) in the main text):

$$f(\mathbf{Y}^*, \mathbf{u_{obs}} \,|\, \theta, \phi, \omega_0, \sigma_m, \sigma_f, \mathbf{u_0}, \mathbf{z}, \mathbf{S}, \mathbf{L})$$
$$= \prod_{i=1}^{M} \left[\left\{ \theta^{u_i}(1-\theta)^{1-u_i} \phi^{y_{i\cdot\cdot}}(1-\phi)^{2n_{i\cdot}-y_{i\cdot\cdot}} \prod_{j=1}^{J} \eta_j(\mathbf{s}_i, u_i)^{n_{ij}} \{(1-\eta_j(\mathbf{s}_i, u_i)) + \eta_j(\mathbf{s}_i, u_i)(1-\phi)^2\}^{K-n_{ij}} \right\}^{z_i} \right]. \tag{B.1}$$

Marginalisation over $\mathbf{u_0}$. Each $u_i \, (\in \mathbf{u})$ is assumed to have a Bernoulli distribution with parameter θ, independent of the other u_j's in $\mathbf{u}$. Now we integrate (B.1) over the missing u_i's.

$$f(\mathbf{Y}^*, \mathbf{u_{obs}} \,|\, \theta, \phi, \omega_0, \sigma_m, \sigma_f, \mathbf{z}, \mathbf{S}, \mathbf{L}) = \sum_{\mathbf{u_0}} f(\mathbf{Y}^*, \mathbf{u_{obs}} \,|\, \theta, \phi, \omega_0, \sigma_m, \sigma_f, \mathbf{u_0}, \mathbf{z}, \mathbf{S}, \mathbf{L}) \, \pi(\mathbf{u_0} \,|\, \mathbf{z}, \theta)$$
$$= \prod_{i: u_i \in \mathbf{u_{obs}}} \Big[\theta^{u_i}(1-\theta)^{1-u_i} \, \phi^{y_{i\cdot\cdot}}(1-\phi)^{2n_{i\cdot}-y_{i\cdot\cdot}}$$
$$\times \prod_{j=1}^{J} \eta_j(\mathbf{s}_i, u_i)^{n_{ij}} \{(1-\eta_j(\mathbf{s}_i, u_i)) + \eta_j(\mathbf{s}_i, u_i)(1-\phi)^2\}^{K-n_{ij}} \Big]^{z_i}$$
$$\times \prod_{i: u_i \in \mathbf{u_0}} \Big[\phi^{y_{i\cdot\cdot}}(1-\phi)^{2n_{i\cdot}-y_{i\cdot\cdot}} \Big\{ \theta \prod_{j=1}^{J} \eta_j(\mathbf{s}_i, 1)^{n_{ij}} \{(1-\eta_j(\mathbf{s}_i, 1)) + \eta_j(\mathbf{s}_i, 1)(1-\phi)^2\}^{K-n_{ij}}$$
$$+ \, (1-\theta) \prod_{j=1}^{J} \eta_j(\mathbf{s}_i, 0)^{n_{ij}} \{(1-\eta_j(\mathbf{s}_i, 0)) + \eta_j(\mathbf{s}_i, 0)(1-\phi)^2\}^{K-n_{ij}} \Big\} \Big]^{z_i}. \tag{B.2}$$

We have assumed that all the individuals with recorded sex category are also detected at least once in one of the detectors (i.e. $y^{(1)}_{i\cdot\cdot} + y^{(2)}_{i\cdot\cdot} > 0$). Whereas among the $\mathbf{u_0}$ individuals, some may be detected and some may not.

Marginalisation over z. Now as mentioned above, each z_i, is assumed to have a Bernoulli prior with parameter ψ, independent of the other z_j's. We integrate (B.2) over $\mathbf{z}$,

$$
\begin{aligned}
& f(\mathbf{Y}^*, \mathbf{u_{obs}} \,|\, \psi, \theta, \phi, \omega_0, \sigma_m, \sigma_f, \mathbf{S}, \mathbf{L}) \\
&\quad = \sum_{\mathbf{z}} f(\mathbf{Y}^*, \mathbf{u_{obs}} \,|\, \theta, \phi, \omega_0, \sigma_m, \sigma_f, \mathbf{z}, \mathbf{S}, \mathbf{L})\, \pi(\mathbf{z} \,|\, \psi) \\
&\quad = \prod_{i:u_i \in \mathbf{u_{obs}}} \Big[\psi\, \theta^{u_i} (1-\theta)^{1-u_i}\, \phi^{y_{i\cdot\cdot}} (1-\phi)^{2n_{i\cdot} - y_{i\cdot\cdot}} \\
&\qquad \times \prod_{j=1}^{J} \eta_j(\mathbf{s}_i, u_i)^{n_{ij}} \{(1-\eta_j(\mathbf{s}_i, u_i)) + \eta_j(\mathbf{s}_i, u_i)(1-\phi)^2\}^{K-n_{ij}} + (1-\psi)\, I(y_{i\cdot\cdot} = 0) \Big] \\
&\qquad \times \prod_{i:u_i \in \mathbf{u_0}} \Big[\psi\, \phi^{y_{i\cdot\cdot}} (1-\phi)^{2n_{i\cdot} - y_{i\cdot\cdot}} \Big\{ \theta \prod_{j=1}^{J} \eta_j(\mathbf{s}_i, 1)^{n_{ij}} \{(1-\eta_j(\mathbf{s}_i, 1)) + \eta_j(\mathbf{s}_i, 1)(1-\phi)^2\}^{K-n_{ij}} \\
&\qquad + (1-\theta) \prod_{j=1}^{J} \eta_j(\mathbf{s}_i, 0)^{n_{ij}} \{(1-\eta_j(\mathbf{s}_i, 0)) + \eta_j(\mathbf{s}_i, 0)(1-\phi)^2\}^{K-n_{ij}} \Big\} + (1-\psi)\, I(y_{i\cdot\cdot} = 0) \Big] \\
&\quad = \prod_{i:u_i \in \mathbf{u_{obs}}} \Big[\psi\, \theta^{u_i} (1-\theta)^{1-u_i}\, \phi^{y_{i\cdot\cdot}} (1-\phi)^{2n_{i\cdot} - y_{i\cdot\cdot}} \\
&\qquad \times \prod_{j=1}^{J} \eta_j(\mathbf{s}_i, u_i)^{n_{ij}} \{(1-\eta_j(\mathbf{s}_i, u_i)) + \eta_j(\mathbf{s}_i, u_i)(1-\phi)^2\}^{K-n_{ij}} \Big] \\
&\qquad \times \prod_{i:u_i \in \mathbf{u_0}} \Big[\psi\, \phi^{y_{i\cdot\cdot}} (1-\phi)^{2n_{i\cdot} - y_{i\cdot\cdot}} \Big\{ \theta \prod_{j=1}^{J} \eta_j(\mathbf{s}_i, 1)^{n_{ij}} \{(1-\eta_j(\mathbf{s}_i, 1)) + \eta_j(\mathbf{s}_i, 1)(1-\phi)^2\}^{K-n_{ij}} \\
&\qquad + (1-\theta) \prod_{j=1}^{J} \eta_j(\mathbf{s}_i, 0)^{n_{ij}} \{(1-\eta_j(\mathbf{s}_i, 0)) + \eta_j(\mathbf{s}_i, 0)(1-\phi)^2\}^{K-n_{ij}} \Big\} + (1-\psi)\, I(y_{i\cdot\cdot} = 0) \Big] \\
&\quad = \prod_{i:u_i \in \mathbf{u_{obs}}} \Big[\psi\, \theta^{u_i} (1-\theta)^{1-u_i}\, \phi^{y_{i\cdot\cdot}} (1-\phi)^{2n_{i\cdot} - y_{i\cdot\cdot}}\, A_i(\mathbf{s}_i, u_i) \Big] \\
&\qquad \times \prod_{i:u_i \in \mathbf{u_0}} \Big[\psi\, \phi^{y_{i\cdot\cdot}} (1-\phi)^{2n_{i\cdot} - y_{i\cdot\cdot}} \Big\{ \theta\, A_i(\mathbf{s}_i, 1) + (1-\theta)\, A_i(\mathbf{s}_i, 0) \Big\} + (1-\psi)\, I(y_{i\cdot\cdot} = 0) \Big],
\end{aligned}
\tag{B.3}
$$

where $A_i(\mathbf{s}_i, u_i) = \prod_{j=1}^{J} \eta_j(\mathbf{s}_i, u_i)^{n_{ij}} \{(1-\eta_j(\mathbf{s}_i, u_i)) + \eta_j(\mathbf{s}_i, u_i)(1-\phi)^2\}^{K-n_{ij}}$, $\pi(\mathbf{z} \,|\, \psi)$ is the density of Bernoulli distribution with parameter ψ.

Marginalisation over S. Each activity centre $\mathbf{s}_i$ is assumed to have a Uniform distribution over the whole state space $\mathbf{S}$, independent of the other $\mathbf{s}_j$'s. Now we integrate (B.3) over $\mathbf{S}$,

$$\begin{aligned}
f(\mathbf{Y}^*, \mathbf{u_{obs}} \,|\, \psi, \theta, \phi, \omega_0, \sigma_m, \sigma_f, \mathbf{L}) &= \int_{\mathbf{S}} f(\mathbf{Y}^*, \mathbf{u_{obs}} \,|\, \psi, \theta, \phi, \omega_0, \sigma_m, \sigma_f, \mathbf{S}, \mathbf{L})\, \pi(\mathbf{S})\, d\mathbf{S} \\
&= \prod_{i: u_i \in \mathbf{u_{obs}}} \Big[\psi \theta^{u_i} (1-\theta)^{1-u_i}\, \phi^{y_{i\cdot\cdot}} (1-\phi)^{2n_{i\cdot} - y_{i\cdot\cdot}} \int_{\mathbf{s}_i} A_i(\mathbf{s}_i, u_i)\, \pi(\mathbf{s}_i)\, d\mathbf{s}_i \Big] \\
&\quad \times \prod_{i: u_i \in \mathbf{u_0}} \Big[\psi\, \phi^{y_{i\cdot\cdot}} (1-\phi)^{2n_{i\cdot} - y_{i\cdot\cdot}} \int_{\mathbf{s}_i} \Big\{ \theta\, A_i(\mathbf{s}_i, 1) + (1-\theta)\, A_i(\mathbf{s}_i, 0) \Big\}\, \pi(\mathbf{s}_i)\, d\mathbf{s}_i + (1-\psi)\, I(y_{i\cdot\cdot} = 0) \Big],
\end{aligned} \tag{B.4}$$

where $\pi(\mathbf{S})$ denotes the density of uniform distribution over the bounded state space $\mathcal{V}$. To compute the integrals in (B.4) over each $\mathbf{s}_i$, first we partition the region $\mathcal{V}$ into a sufficiently fine grid and then evaluate a Riemann sum, since each of those integrals can not be expressed in closed form. Assume $\{\mathbf{s}^{(l)} : l = 1, 2, \ldots, n_G\}$ are the pixel centres of one such fine grid over $\mathcal{V}$. Then (B.4) becomes

$$\begin{aligned}
f(\mathbf{Y}^*, \mathbf{u_{obs}} \,|\, \psi, \theta, \phi, \omega_0, \sigma_m, \sigma_f, \mathbf{L}) &= \int_{\mathbf{S}} f(\mathbf{Y}^*, \mathbf{u_{obs}} \,|\, \psi, \theta, \phi, \omega_0, \sigma_m, \sigma_f, \mathbf{S}, \mathbf{L})\, \pi(\mathbf{S})\, d\mathbf{S} \\
&\approx \prod_{i: u_i \in \mathbf{u_{obs}}} \Big[\psi \theta^{u_i} (1-\theta)^{1-u_i}\, \phi^{y_{i\cdot\cdot}} (1-\phi)^{2n_{i\cdot} - y_{i\cdot\cdot}} \Big\{ \frac{1}{n_G} \sum_{l=1}^{n_G} A_i(\mathbf{s}^{(l)}, u_i) \Big\} \Big] \\
&\quad \times \prod_{i: u_i \in \mathbf{u_0}} \Big[\psi\, \phi^{y_{i\cdot\cdot}} (1-\phi)^{2n_{i\cdot} - y_{i\cdot\cdot}} \Big\{ \frac{1}{n_G} \sum_{l=1}^{n_G} \Big(\theta\, A_i(\mathbf{s}^{(l)}, 1) + (1-\theta)\, A_i(\mathbf{s}^{(l)}, 0) \Big) \Big\} + (1-\psi)\, I(y_{i\cdot\cdot} = 0) \Big] \\
&= \prod_{i: u_i \in \mathbf{u_{obs}}} \Big[\psi \theta^{u_i} (1-\theta)^{1-u_i}\, \phi^{y_{i\cdot\cdot}} (1-\phi)^{2n_{i\cdot} - y_{i\cdot\cdot}} \Big\{ \frac{1}{n_G} \sum_{l=1}^{n_G} \prod_{j=1}^{J} \eta_j(\mathbf{s}^{(l)}, u_i)^{n_{ij}}\, \{(1 - \eta_j(\mathbf{s}^{(l)}, u_i)) \\
&\quad + \eta_j(\mathbf{s}^{(l)}, u_i)(1-\phi)^2\}^{K - n_{ij}} \Big\} \Big] \\
&\quad \times \prod_{i: u_i \in \mathbf{u_0}} \Big[\psi\, \phi^{y_{i\cdot\cdot}} (1-\phi)^{2n_{i\cdot} - y_{i\cdot\cdot}} \Big\{ \frac{1}{n_G} \sum_{l=1}^{n_G} \Big(\theta \prod_{j=1}^{J} \eta_j(\mathbf{s}^{(l)}, 1)^{n_{ij}}\, \{(1 - \eta_j(\mathbf{s}^{(l)}, 1)) + \eta_j(\mathbf{s}^{(l)}, 1)(1-\phi)^2\}^{K - n_{ij}} \\
&\quad + (1-\theta) \prod_{j=1}^{J} \eta_j(\mathbf{s}^{(l)}, 0)^{n_{ij}}\, \{(1 - \eta_j(\mathbf{s}^{(l)}, 0)) + \eta_j(\mathbf{s}^{(l)}, 0)(1-\phi)^2\}^{K - n_{ij}} \Big) \Big\} + (1-\psi)\, I(y_{i\cdot\cdot} = 0) \Big].
\end{aligned} \tag{B.5}$$

Observe that, when $y_{i\cdot\cdot} = 0$, $A_i(\mathbf{s}^{(l)}, 1) = \prod_{j=1}^{J} \{(1 - \eta_j(\mathbf{s}^{(l)}, 1)) + \eta_j(\mathbf{s}^{(l)}, 1)(1-\phi)^2\}^J$, $A_i(\mathbf{s}^{(l)}, 0) = \prod_{j=1}^{J} \{(1 - \eta_j(\mathbf{s}^{(l)}, 0)) + \eta_j(\mathbf{s}^{(l)}, 0)(1-\phi)^2\}^J$.

B.2 Integrated likelihood under M_2

The joint density of $\mathbf{Y}^* := (\mathbf{Y}^{(\mathbf{1})}, \mathbf{Y}^{(\mathbf{2*})}) = ((y_{ijk}^{(1)}, y_{ijk}^{(2*)}))$ and $\mathbf{u} = (u_1, \dots, u_M)'$ under M_2 is the following (also given as model likelihood (2.2) in the main text):

$$f_{\mathrm{R}}(\mathbf{Y}^*, \mathbf{u_{obs}} \,|\, \theta, p_0, \sigma_m, \sigma_f, \mathbf{u_0}, \mathbf{z}, \mathbf{S}, \mathbf{L}) = \prod_{i=1}^{M} \left[\left\{ \theta^{u_i}(1-\theta)^{1-u_i} \prod_{j=1}^{J} p_j(\mathbf{s}_i, u_i)^{y_{ij\cdot}} (1 - p_j(\mathbf{s}_i, u_i))^{2K - y_{ij\cdot}} \right\}^{z_i} \right], \tag{B.6}$$

The corresponding integrated likelihood is obtained by integrating (B.6) with respect to $\mathbf{u}_0$, $\mathbf{z}$, $\mathbf{S}$:

$$\begin{aligned}
&f_{\mathrm{R}}(\mathbf{Y}^*, \mathbf{u_{obs}} \,|\, \psi, \theta, p_0, \sigma_m, \sigma_f, \mathbf{L}) \\
&= \int_{\mathbf{S}} \sum_{\mathbf{u_0}, \mathbf{z}} f(\mathbf{Y}^*, \mathbf{u_{obs}} \,|\, \psi, \theta, p_0, \sigma_m, \sigma_f, \mathbf{u_0}, \mathbf{z}, \mathbf{S}, \mathbf{L})\, \pi(\mathbf{u_0} \,|\, \mathbf{z}, \theta)\, \pi(\mathbf{z} \,|\, \psi)\, \pi(\mathbf{S})\, d\mathbf{S} \\
&\approx \prod_{i: u_i \in \mathbf{u_{obs}}} \left[\psi \theta^{x_i} (1-\theta)^{1-x_i} \left\{ \frac{1}{n_G} \sum_{l=1}^{n_G} B_i(\mathbf{s}^{(l)}, u_i) \right\} \right] \\
&\quad \times \prod_{i: u_i \in \mathbf{u_0}} \left[\psi \left\{ \frac{1}{n_G} \sum_{l=1}^{n_G} \Big(\theta\, B_i(\mathbf{s}^{(l)}, 1) + (1-\theta)\, B_i(\mathbf{s}^{(l)}, 0) \Big) \right\} + (1-\psi)\, I(y_{i\cdot\cdot} = 0) \right],
\end{aligned} \tag{B.7}$$

where $B_i(\mathbf{s}, u) = \prod_{j=1}^{J} p_j(\mathbf{s}, u)^{y_{ij\cdot}} (1 - p_j(\mathbf{s}, u))^{2K - y_{ij\cdot}}$, $\pi(\mathbf{z} \,|\, \psi)$ is the density of Bernoulli distribution with parameter ψ, $\pi(\mathbf{S})$ denotes the density of uniform distribution over the bounded state space $\mathcal{V}$.

B.3 Integrated likelihood under M_3

The joint density of $\mathbf{Y}^* := (\mathbf{Y}^{(1)}, \mathbf{Y}^{(2*)}) = ((y_{ijk}^{(1)}, y_{ijk}^{(2*)}))$ under M_3 is the following (also given as model likelihood (2.3) in the main text):

$$\begin{aligned} & f(\mathbf{Y}^* \mid \phi, \omega_0, \sigma, \mathbf{z}, \mathbf{S}, \mathbf{L}) \\ & = \prod_{i=1}^{M} \left\{ \phi^{y_{i\cdot\cdot}} (1-\phi)^{2n_{i\cdot} - y_{i\cdot\cdot}} \prod_{j=1}^{J} \eta_j(\mathbf{s}_i)^{n_{ij}} \{(1-\eta_j(\mathbf{s}_i)) + \eta_j(\mathbf{s}_i)(1-\phi)^2\}^{K-n_{ij}} \right\}^{z_i}. \end{aligned} \tag{B.8}$$

After integrating the above likelihood (B.8) over $\mathbf{z}$ and $\mathbf{S}$ with respect to their priors, we obtain

$$\begin{aligned} & f(\mathbf{Y}^* \mid \phi, \omega_0, \sigma, \mathbf{L}) \\ & = \int_{\mathbf{S}} \sum_{\mathbf{z}} f(\mathbf{Y}^* \mid \phi, \omega_0, \sigma, \mathbf{z}, \mathbf{S}, \mathbf{L})\, \pi(\mathbf{z} \mid \psi)\, \pi(\mathbf{S})\, d\mathbf{S} \\ & \approx \prod_{i: y_{i\cdot\cdot} > 0} \left[\psi\, \phi^{y_{i\cdot\cdot}} (1-\phi)^{2n_{i\cdot} - y_{i\cdot\cdot}} \left\{ \frac{1}{n_G} \sum_{l=1}^{n_G} \prod_{j=1}^{J} \eta_j(\mathbf{s}^{(l)})^{n_{ij}} \{(1-\eta_j(\mathbf{s}^{(l)})) + \eta_j(\mathbf{s}^{(l)})(1-\phi)^2\}^{K-n_{ij}} \right\} \right] \\ & \quad \times \prod_{i: y_{i\cdot\cdot} = 0} \left[\psi\, \phi^{y_{i\cdot\cdot}} (1-\phi)^{2n_{i\cdot} - y_{i\cdot\cdot}} \left\{ \frac{1}{n_G} \sum_{l=1}^{n_G} \prod_{j=1}^{J} \eta_j(\mathbf{s}^{(l)})^{n_{ij}} \{(1-\eta_j(\mathbf{s}^{(l)})) + \eta_j(\mathbf{s}^{(l)})(1-\phi)^2\}^{K-n_{ij}} \right\} \right. \\ & \qquad\qquad \left. + (1-\psi)\, I(y_{i\cdot\cdot} = 0) \right] \\ & = \prod_{i: y_{i\cdot\cdot} > 0} \left[\psi\, \phi^{y_{i\cdot\cdot}} (1-\phi)^{2n_{i\cdot} - y_{i\cdot\cdot}} \left\{ \frac{1}{n_G} \sum_{l=1}^{n_G} A_i(\mathbf{s}^{(l)}) \right\} \right] \\ & \quad \times \prod_{i: y_{i\cdot\cdot} = 0} \left[\psi\, \phi^{y_{i\cdot\cdot}} (1-\phi)^{2n_{i\cdot} - y_{i\cdot\cdot}} \left\{ \frac{1}{n_G} \sum_{l=1}^{n_G} A_i(\mathbf{s}^{(l)}) \right\} + (1-\psi) \right], \end{aligned} \tag{B.9}$$

where $A_i(\mathbf{s}) = \prod_{j=1}^{J} \eta_j(\mathbf{s})^{n_{ij}} \{(1-\eta_j(\mathbf{s})) + \eta_j(\mathbf{s})(1-\phi)^2\}^{K-n_{ij}}$, $\pi(\mathbf{z} \mid \psi)$ is the density of Bernoulli distribution with parameter ψ, $\pi(\mathbf{S})$ denotes the density of uniform distribution over the bounded state space $\mathcal{V}$.

B.4 Integrated likelihood under M_4

The joint density of $\mathbf{Y}^* := (\mathbf{Y}^{(1)}, \mathbf{Y}^{(2*)}) = ((y_{ijk}^{(1)}, y_{ijk}^{(2*)}))$ under M_4 is the following (also given as model likelihood (2.4) in the main text):

$$f_{\mathrm{R}}(\mathbf{Y}^* \,|\, p_0, \sigma, \mathbf{z}, \mathbf{S}, \mathbf{L}) = \prod_{i=1}^{M} \prod_{j=1}^{J} \left\{ p_j(\mathbf{s}_i)^{y_{ij\cdot}} (1 - p_j(\mathbf{s}_i))^{2K - y_{ij\cdot}} \right\}^{z_i}, \tag{B.10}$$

The integrated likelihood in the absence of individual covariate on sex category $\mathbf{u}$ is obtained by integrating (B.10) with respect to $\mathbf{z}$, $\mathbf{S}$, and is given by

$$\begin{aligned} f_{\mathrm{R}}(\mathbf{Y}^* \,|\, \psi, p_0, \sigma, \mathbf{L}) &= \int_{\mathbf{S}} \sum_{\mathbf{z}} f_{\mathrm{R}}(\mathbf{Y}^* \,|\, \psi, p_0, \sigma, \mathbf{z}, \mathbf{S}, \mathbf{L})\, \pi(\mathbf{z} \,|\, \psi)\, \pi(\mathbf{S})\, d\mathbf{S} \\ &\approx \prod_{i: y_{i\cdot\cdot} > 0} \left[\psi \left\{ \frac{1}{n_G} \sum_{l=1}^{n_G} B_i(\mathbf{s}^{(l)}) \right\} \right] \times \prod_{i: y_{i\cdot\cdot} = 0} \left[\psi \left\{ \frac{1}{n_G} \sum_{l=1}^{n_G} B_i(\mathbf{s}^{(l)}) \right\} + (1 - \psi) \right], \end{aligned} \tag{B.11}$$

where $B_i(\mathbf{s}) = \prod_{j=1}^{J} p_j(\mathbf{s})^{y_{ij\cdot}} (1 - p_j(\mathbf{s}))^{2K - y_{ij\cdot}}$, $\pi(\mathbf{z} \,|\, \psi)$ is the density of Bernoulli distribution with parameter ψ and $\pi(\mathbf{S})$ denotes the density of uniform distribution over the bounded state space $\mathcal{V}$.

C Transformed prior distributions and tuning densities

Under our model settings, $\boldsymbol{\mu}$ denotes the collection of all parameters and latent variables for each model as a generic notation. Specifically, we let $\boldsymbol{\mu} = (\boldsymbol{\mu_p}, \boldsymbol{\mu_s})$, where $\boldsymbol{\mu_p}$ is the collection of scalar parameters and $\boldsymbol{\mu_s}$ is the collection of all latent variables. As indicated in Section 3.1 of the main text, first we transform the components of $\boldsymbol{\mu_p}$ so that the transformed parameter space is the entire Euclidean space and also to get a better mixing during the MCMC run. The transformations on the components of $\boldsymbol{\mu_p}$, i.e., the parameters ψ, θ, ϕ, ω_0, p_0, σ, σ_m, σ_f are as follows: $\psi^* = \mathrm{logit}(\psi)$, $\theta^* = \mathrm{logit}(\theta)$, $\phi^* = \mathrm{logit}(\phi)$, $\omega_0^* = \mathrm{logit}(\omega_0)$, $p_0^* = \mathrm{logit}(p_0)$, $\sigma^* = \log(\sigma)$, $\sigma_m^* = \log(\sigma_m)$, $\sigma_f^* = \log(\sigma_f)$. We denote the set of transformed parameters as $\boldsymbol{\mu_p^*} = h(\boldsymbol{\mu_p})$ and the transformed prior

by $\pi^*(\boldsymbol{\mu}_{\boldsymbol{p}}^*)$ with domain as the Euclidean space.

Transformed prior distributions.

The prior distribution on the parameters ψ, θ, ϕ, ω_0, p_0, σ, σ_m and σ_f are given in Table 1. The transformed prior on ψ^*, θ^*, ϕ^*, ω_0^*, p_0^*,, σ^*, σ_m^* and σ_f^* are

$$\pi^*(\psi^*) = \pi\big(h^{-1}(\psi^*)\big) \mid J \mid = \frac{\exp(\psi^*)}{(1+\exp\big(\psi^*)\big)^2}, \quad \pi^*(\theta^*) = \pi\big(h^{-1}(\theta^*)\big) \mid J \mid = \frac{\exp(\theta^*)}{(1+\exp\big(\theta^*)\big)^2},$$
$$\pi^*(\phi^*) = \pi\big(h^{-1}(\phi^*)\big) \mid J \mid = \frac{\exp(\phi^*)}{(1+\exp\big(\phi^*)\big)^2}, \quad \pi^*(\omega_0^*) = \pi\big(h^{-1}(\omega_0^*)\big) \mid J \mid = \frac{\exp(\omega_0^*)}{(1+\exp\big(\omega_0^*)\big)^2},$$
$$\pi^*(p_0^*) = \pi\big(h^{-1}(p_0^*)\big) \mid J \mid = \frac{\exp(p_0^*)}{(1+\exp\big(p_0^*)\big)^2}, \quad \pi^*(\sigma^*) = \pi\big(h^{-1}(\sigma^*)\big) \mid J \mid = \frac{\exp(\sigma^*)}{R}$$
$$\pi^*(\sigma_m^*) = \pi\big(h^{-1}(\sigma_m^*)\big) \mid J \mid = \frac{\exp(\sigma_m^*)}{R}, \quad \pi^*(\sigma_f^*) = \pi\big(h^{-1}(\sigma_f^*)\big) \mid J \mid = \frac{\exp(\sigma_f^*)}{R},$$

where $\mid J \mid$ denotes the Jacobian of transformation and $h^{-1}(\cdot)$ denotes the inverse transformation of the parameter.

Tuning density.

We have carried out the computation of Gelfand-Dey estimator using different choices of $g(\boldsymbol{\mu}_{\boldsymbol{p}}^*)$ to assess the sensitivity of the resulting estimates (see Section 2.2.1). First, density of the multivariate normal distribution $\mathcal{N}(\hat{\boldsymbol{\mu}}_{\boldsymbol{p}}^*, \hat{\Sigma})$ with mean $\hat{\boldsymbol{\mu}}_{\boldsymbol{p}}^*$ and covariance matrix $\hat{\Sigma}$ is chosen. $\hat{\boldsymbol{\mu}}_{\boldsymbol{p}}^*$ and $\hat{\Sigma}$ are computed by using the MCMC draws $\{\boldsymbol{\mu}_{\boldsymbol{p}}{}^{*(d)} : d = 1, \ldots, N_{iter}\}$. The mean parameter $\hat{\boldsymbol{\mu}}_{\boldsymbol{p}}^* = (\hat{\mu}_{p1}^*, \hat{\mu}_{p2}^*, \ldots)$ has components $\hat{\mu}_{pi}^* = \frac{1}{N_{iter}} \sum_{d=1}^{N_{iter}} \mu_{pi}^{*(d)}$. The entries of the covariance matrix $\hat{\Sigma}$ can be obtained by using the MCMC sample as

$$\hat{\Sigma}_{ii} = \frac{1}{N_{iter}-1} \sum_{d=1}^{N_{iter}} (\mu_{pi}^{*(d)} - \hat{\mu}_{pi}^*)^2, \ \hat{\Sigma}_{ij} = \frac{1}{N_{iter}-1} \sum_{d=1}^{N_{iter}} (\mu_{pi}^{*(d)} - \hat{\mu}_{pi}^*)\,(\mu_{pj}^{*(d)} - \hat{\mu}_{pj}^*).$$

Additionally, we have used multivariate-t densities for $g(\boldsymbol{\mu}_{\boldsymbol{p}}^*)$ with mean parameter $\boldsymbol{\mu}_{\boldsymbol{p}}^*$ and scale matrix $\hat{\Sigma}$ using different degrees of freedom: 10, 100, 500, 1000, 10000. A truncated normal density has also been tried for $g(\boldsymbol{\mu}_{\boldsymbol{p}}^*)$ following the suggestion of Geweke (1999). The parameters of the truncated normal density $\hat{\boldsymbol{\mu}}_{\boldsymbol{p}}^*, \hat{\Sigma}$ are obtained as above.

Table 1: Prior distributions and transformations of model parameters and latent variables that are used for the MCMC algorithms. Here $|\,J\,|$ denotes the Jacobian of transformation and $h^{-1}(\cdot)$ denotes the inverse transformation of the parameter.

**Parameter**	**Prior**	**Transformed parameter**	**Transformed prior pdf**	**Corresponding models**
ϕ	Uniform(0, 1)	$\phi^* = \text{logit}(\phi)$	$\dfrac{\exp(\phi^*)}{\left(1+\exp\left(\phi^*\right)\right)^2}$	M_1, M_3
ω_0	Uniform(0, 1)	$\omega_0^* = \text{logit}(\omega_0)$	$\dfrac{\exp(\omega_0^*)}{\left(1+\exp\left(\omega_0^*\right)\right)^2}$	M_1, M_3
p_0	Uniform(0, 1)	$p_0^* = \text{logit}(p_0)$	$\dfrac{\exp(p_0^*)}{\left(1+\exp\left(p_0^*\right)\right)^2}$	M_2, M_4
σ	Uniform(0, R)	$\sigma^* = \log(\sigma)$	$\dfrac{\exp(\sigma^*)}{R}$	M_3, M_4
σ_m	Uniform(0, R)	$\sigma_m^* = \log(\sigma_m)$	$\dfrac{\exp(\sigma_m^*)}{R}$	M_1, M_2
σ_f	Uniform(0, R)	$\sigma_f^* = \log(\sigma_f)$	$\dfrac{\exp(\sigma_f^*)}{R}$	M_1, M_2
u_i	Bernoulli(θ)			M_1, M_2
θ	Uniform(0, 1)	$\theta^* = \text{logit}(\theta)$	$\dfrac{\exp(\theta^*)}{\left(1+\exp\left(\theta^*\right)\right)^2}$	M_1, M_2
z_i	Bernoulli(ψ)			M_1, M_2, M_3, M_4
ψ	Uniform(0, 1)	$\psi^* = \text{logit}(\psi)$	$\dfrac{\exp(\psi^*)}{\left(1+\exp\left(\psi^*\right)\right)^2}$	M_1, M_2, M_3, M_4
$\mathbf{L}$	Uniform distribution over the permutation space of $\{1,\ldots,M\}$	-<br>-	-<br>-	M_1, M_2, M_3, M_4
$\mathbf{s}_i = (s_{i1}, s_{i2})'$	Uniform($\mathcal{V}$)	-	-	M_1, M_2, M_3, M_4

Then for some $\alpha \in (0,1)$, we define the set $\hat{\Theta}$ as follows:

$$\hat{\Theta} = \left\{\boldsymbol{\mu}_{\boldsymbol{p}}^* \,:\, (\boldsymbol{\mu}_{\boldsymbol{p}}^* - \hat{\boldsymbol{\mu}}_{\boldsymbol{p}}^*)'\,\hat{\Sigma}^{-1}\,(\boldsymbol{\mu}_{\boldsymbol{p}}^* - \hat{\boldsymbol{\mu}}_{\boldsymbol{p}}^*) \;\leq\; \chi^2_{r}(\alpha)\right\}$$

and we take

$$g(\boldsymbol{\mu}_{\boldsymbol{p}}^*) = \alpha^{-1}(2\pi)^{-r/2}|\hat{\Sigma}|^{-1/2}\exp\left[(\boldsymbol{\mu}_{\boldsymbol{p}}^* - \hat{\boldsymbol{\mu}}_{\boldsymbol{p}}^*)'\,\hat{\Sigma}^{-1}\,(\boldsymbol{\mu}_{\boldsymbol{p}}^* - \hat{\boldsymbol{\mu}}_{\boldsymbol{p}}^*)\right] I(\boldsymbol{\mu}_{\boldsymbol{p}}^* \in \hat{\Theta}),$$

where $\chi^2_{r}(\alpha)$ denotes $100\,\alpha\%$ quantile of χ^2_{r} distribution with r denoting the dimension of $\boldsymbol{\mu}_{\boldsymbol{p}}^*$. We have carried out the computations for three values of α: 0.9, 0.95 and 0.99.

Note that, in the integrated likelihood (IL) approximation approach for computing Bayes factors, we assume $g(\boldsymbol{\mu}_{\boldsymbol{p}}^*, \mathbf{L}) = g_1(\boldsymbol{\mu}_{\boldsymbol{p}}^*)\,g_2(\mathbf{L})$. We take $g_1(\boldsymbol{\mu}_{\boldsymbol{p}}^*)$ from the above choices in MAP approximation approach and take the prior density $\pi(\mathbf{L})$ as the tuning density $g_2(\mathbf{L})$.

D Steps of MCMC sampling for SCR models with partially identified individuals

Steps of MCMC sampling

The joint posterior density of the parameter $\boldsymbol{\mu} := \{\psi, \theta, \phi, \omega_0, \sigma_m, \sigma_f, \mathbf{u_0}, \mathbf{z}, \mathbf{S}, \mathbf{L}\}$ under a model M_i is obtained by using the corresponding model likelihood and the prior distributions of the parameters and latent variables, $i = 1, 2, 3, 4$. For instance, the joint posterior density of $\boldsymbol{\mu}$ under M_1 is given below (using the model likelihood (**??**) from the main text and the prior distributions mentioned in Table 1):

$$\begin{aligned}
&\pi(\boldsymbol{\mu} \,|\, \mathbf{Y}^*, \mathbf{u_{obs}}) \propto f(\mathbf{Y}^*, \mathbf{u_{obs}} \,|\, \boldsymbol{\mu})\, \pi(\mathbf{z} \,|\, \psi)\, \pi(\mathbf{u_0} \,|\, \mathbf{z}, \theta)\, \pi(\psi, \theta, \phi, \omega_0, \sigma_m, \sigma_f, \mathbf{S}, \mathbf{L}) \\
&= \prod_{i=1}^{M} \Big[\Big\{ \psi \theta^{u_i} (1-\theta)^{1-u_i}\, \phi^{y_{i\cdot\cdot}} (1-\phi)^{2n_{i\cdot} - y_{i\cdot\cdot}} \prod_{j=1}^{J} \eta_j(\mathbf{s}_i, u_i)^{n_{ij}} \{(1 - \eta_j(\mathbf{s}_i, u_i)) \\
&\quad + \eta_j(\mathbf{s}_i, u_i)(1-\phi)^2\}^{K - n_{ij}} \Big\}^{z_i} (1-\psi)^{1-z_i} \Big] \times \pi(\psi, \theta, \phi, \omega_0, \sigma_m, \sigma_f, \mathbf{S}, \mathbf{L}), \qquad \text{(D.1)}
\end{aligned}$$

where $y_{ij\cdot} = y_{ij\cdot}^{(1)} + y_{ij\cdot}^{(2*)}$, $y_{i\cdot\cdot} = y_{i\cdot\cdot}^{(1)} + y_{i\cdot\cdot}^{(2*)}$, $n_{ij} = \sum_{k=1}^{K} I(y_{ijk}^{(1)} + y_{ijk}^{(2*)} > 0)$, $n_{i\cdot} = \sum_{j=1}^{J} n_{ij}$ and $\pi(\psi, \theta, \phi, \omega_0, \sigma_m, \sigma_f, \mathbf{S}, \mathbf{L})$ is the joint prior density for the parameters $\psi, \theta, \phi, \omega_0, \sigma_m, \sigma_f, \mathbf{S}, \mathbf{L}$. The other posterior densities can be obtained similarly.

Metropolis-within-Gibbs is easy to implement and is quite flexible in this modelling set up. Since parameters are updated sequentially, it is easy for investigators to follow and even modify specifications such as jumping probabilities etc. Here, Metropolis within Gibbs sampling is performed to obtain MCMC chains for the components of $\boldsymbol{\mu}$ (viz., ψ, θ, ϕ, ω_0, σ_m, σ_f, $\mathbf{u_0}$, $\mathbf{z}$, $\mathbf{S}$, $\mathbf{L}$ for model M_1). The transformations and the assumed prior distributions are given in Table 1. Next we give the steps of each iteration of the MCMC analysis.

1. **Initialisation.** Initialise each of the components of $\boldsymbol{\mu}$. While initialising $\mathbf{L}$, we should take care of the fact that no mismatch takes place with respect to the sexes, i.e., we should avoid linking male (or female) individual from detector 2 to female (or male) individual from detector 1.

2. **Augmentation.** Augment all-zero detection histories to $\mathbf{Y}^{(1)}_{\mathbf{obs}}$ and $\mathbf{Y}^{(2)}_{\mathbf{obs}}$ for making them of dimension $M \times J \times K$. We denote them $\mathbf{Y}^{(1)}$ and $\mathbf{Y}^{(2)}$.

3. **Re-ordering.** Given the matching vector $\mathbf{L}$, we re-order the rows of $\mathbf{Y}^{(2)}$ so that they are matched properly with $\mathbf{Y}^{(1)}$. We denote this ordered right side data set as $\mathbf{Y}^{(2*)}$.

4. **Update the movement scale parameter(s).**

   (a) Under M_1 or M_2, we have σ^*_m and σ^*_f as the movement scale parameters and are updated under each of these two models similarly. For model M_1, the full conditional of σ^*_m is obtained from (D.1) and is of a non-standard form

$$\pi(\sigma^*_m|\mathbf{Y}^*, \boldsymbol{\mu}_{\text{rest}}) \\ \propto \pi(\sigma^*_m) \prod_{i:u_i=1} \prod_{j=1}^{J} \eta_j(\mathbf{s}_i, u_i)^{z_i n_{ij}} \left\{(1-\eta_j(\mathbf{s}_i, u_i)) + \left(1 - \frac{\exp(\phi^*)}{1+\exp(\phi^*)}\right)^2 \eta_j(\mathbf{s}_i, u_i)\right\}^{z_i(K-n_{ij})},$$

   where $\boldsymbol{\mu}_{\text{rest}}$ denotes all the remaining parameters other than σ^*_m. Here a normal proposal is assumed for σ^*_m and is updated using a Metropolis algorithm. The parameters of this proposal distribution at t^{th} iteration are $\sigma^{*(t-1)}_m$ and τ^2_m, where $\sigma^{*(t-1)}_m$ is the $(t-1)^{\text{th}}$ element in the Markov chain of σ^*_m. The movement scale parameter for female individuals σ^*_f is updated in a similar manner.

   (b) Under M_3 or M_4, we have σ^* as the only movement scale parameters and is updated under each of these two models similarly. For model M_3, the full conditional of σ^* is obtained from the corresponding joint posterior density of $\boldsymbol{\mu}$ and is of a non-standard form

$$\pi(\sigma^* \,|\, \mathbf{Y}^*, \boldsymbol{\mu}_{\text{rest}}) \propto \pi^*(\sigma^*) \prod_{i=1}^{M} \prod_{j=1}^{J} \eta_j(\mathbf{s}_i)^{z_i n_{ij}} \left\{(1-\eta_j(\mathbf{s}_i)) + \left(1 - \frac{\exp(\phi^*)}{1+\exp(\phi^*)}\right)^2 \eta_j(\mathbf{s}_i)\right\}^{z_i(K-n_{ij})}.$$

   where $\boldsymbol{\mu}_{\text{rest}}$ denotes all the remaining parameters other than σ^*. Here a normal proposal is assumed for σ^* and is updated using a Metropolis algorithm. The parameters of this proposal distribution at t^{th} iteration are $\sigma^{*(t-1)}$ and τ^2, where $\sigma^{*(t-1)}$ is the $(t-1)^{\text{th}}$ element in the Markov chain of σ^*.

5. **Update each $u_i \in \mathbf{u_0}$ (only for M_1 and M_2).** When $z_i = 1$, the full conditional

distribution of u_i ($\in \mathbf{u_0}$) is the Bernoulli distribution with parameter θ_{0i}. Under model M_1, θ_{0i} takes the following form

$$\theta_{0i} = \frac{\theta\, A_i(\mathbf{s}_i, 1)}{\theta\, A_i(\mathbf{s}_i, 1) + (1-\theta)\, A_i(\mathbf{s}_i, 0)}$$

where $A_i(\mathbf{s}_i, u_i) = \prod_{j=1}^{J} \eta_j(\mathbf{s}_i, u_i)^{n_{ij}} \left\{(1-\eta_j(\mathbf{s}_i, u_i)) + \left(1 - \frac{\exp(\phi^*)}{1+\exp(\phi^*)}\right)^2 \eta_j(\mathbf{s}_i, u_i)\right\}^{K-n_{ij}}$. Note that the full conditional of u_i is independent of any other u_a's ($a \neq i$), $u_i, u_a \in \mathbf{u}$. When $u_i \notin \mathbf{u_0}$ or $z_i = 0$, we don't require to update u_i. The latent variable u_i is updated in a similar manner under M_2.

6. **Update θ (only for M_1 and M_2).** The full conditional distribution of θ is $Beta(\sum_{i=1}^{M} z_i u_i + 1, \sum_{i=1}^{M} z_i(1-u_i) + 1)$ under both M_1 and M_2.

7. **Update each z_i.** For all the detected individuals, i.e., i's such that $y_{i\cdot\cdot}^{(1)} + y_{i\cdot\cdot}^{(2*)} > 0$, the full conditional of z_i has a degenerate distribution at the value 1, denoted by

$$\pi(z_i \,|\, y_{i\cdot\cdot}^{(1)}, y_{i\cdot\cdot}^{(2*)}, y_{i\cdot\cdot}^{(1)} + y_{i\cdot\cdot}^{(2*)} > 0, \boldsymbol{\mu}_{\text{rest}}) = I(z_i = 1)$$

where $\boldsymbol{\mu}_{\text{rest}}$ denotes all the remaining parameters other than z_i. For the undetected individuals with all zero detection histories (i.e., $y_{i\cdot\cdot}^{(1)} = 0$ and $y_{i\cdot\cdot}^{(2*)} = 0$), the full conditional distribution of z_i is Bernoulli with parameter ψ_{i0}. Under M_1, ψ_{i0} has the following expression

$$\psi_{i0} = \frac{\psi\, \theta^{u_i}(1-\theta)^{1-u_i} \prod_{j=1}^{J} \left\{(1-\eta_j(\mathbf{s}_i, u_i)) + \left(1 - \frac{\exp(\phi^*)}{1+\exp(\phi^*)}\right)^2 \eta_j(\mathbf{s}_i, u_i)\right\}^K}{\psi\, \theta^{u_i}(1-\theta)^{1-u_i} \prod_{j=1}^{J} \left\{(1-\eta_j(\mathbf{s}_i, u_i)) + \left(1 - \frac{\exp(\phi^*)}{1+\exp(\phi^*)}\right)^2 \eta_j(\mathbf{s}_i, u_i)\right\}^K + (1-\psi)}.$$

Note that the full conditional distribution of z_i is independent of any other z_j's ($j \neq i$). The full conditional distribution of z_i can be obtained similarly for the other models.

8. **Update ψ.** The full conditional distribution of ψ is $Beta(\sum_{i=1}^{M} z_i + 1, M - \sum_{i=1}^{M} z_i + 1)$.

9. **Update each $\mathbf{s}_i$.** For model M_1, the full conditional distribution for $\mathbf{s}_i$ is of a

non-standard form with density

$$\pi(\mathbf{s}_i \,|\, \mathbf{Y}^*, \boldsymbol{\mu}_{\text{rest}}) \propto \pi(\mathbf{s}_i) \prod_{j=1}^{J} \eta_j(\mathbf{s}_i, u_i)^{z_i n_{ij}} \Big\{(1-\eta_j(\mathbf{s}_i, u_i)) + \Big(1 - \frac{\exp(\phi^*)}{1+\exp(\phi^*)}\Big)^2 \eta_j(\mathbf{s}_i, u_i)\Big\}^{z_i(K-n_{ij})},$$

where $\boldsymbol{\mu}_{\text{rest}}$ denotes all the remaining parameters other than $\mathbf{s}_i$. Each $\mathbf{s}_i$ can be simulated using a random walk Metropolis algorithm. A normal proposal is assumed for $\mathbf{s}_i$. The parameters of this proposal distribution at t^{th} iteration are $\mathbf{s}_i^{(t-1)}$ and τ_s^2, where $\mathbf{s}_i^{(t-1)}$ is the $(t-1)^{\text{th}}$ element in the Markov chain of $\mathbf{s}_i$. Note that the full conditional distribution of $\mathbf{s}_i$ is independent of any other $\mathbf{s}_j$'s $(j \neq i)$. Each $\mathbf{s}_i$ can be updated similarly for other models.

10. **Update L.**

   (a) We assume a Uniform prior over the permutation space of $\mathbf{L}$. Observe that, each individual from detector 2 (ID2) can be linked with only one from the list of the individuals from detector 1 (ID1s) (of the same sex). The technique to update $\mathbf{L}$ is given below.

   (b) We start by selecting one ID2, say i and get $\mathrm{L}_i = l$ (say), the ID1 (and also the corresponding $\mathbf{s}_l$) to whom it was linked in the previous iteration. The activity centres $\mathbf{S}$ and data on sex $\mathbf{u}$ are in the same order as ID1's.

   (c) Then we find the set of (real) individual indices of the same sex as ID1 l which is also the candidate set to select a new ID1 for ID2 i. We then calculate the weights proportional to $b_{l,a} = \exp(-\frac{1}{2\sigma_L^2}\|\mathbf{s}_l - \mathbf{s}_a\|^2)$ for each individual in this set, where $\mathbf{s}_a$ is the the location of the activity centre for ID1 a and σ_L is a tuning parameter. A good choice for σ_L can be σ_m or σ_f based on whether u_l is 1 or 0. We randomly draw one ID1 from this candidate set, say l^*, according to these weights.

   (d) This is the swapping step. We find the ID2 who is linked with ID1 l^* and denote it by i^*. Now we link ID2 i^* with ID1 l and link ID2 i with ID1 l^*. Consequently, we have $\mathrm{L}_{i^*} = l$ and $\mathrm{L}_i = l^*$. We also find the set of (real) activity centres with same sex as ID1 j^* and calculate the weights $b_{l^*,a}^{back} =$

$\exp(-\frac{1}{2\sigma_L^2}\|\mathbf{s}_{l^*} - \mathbf{s}_a\|^2)$ for each individual in this set.

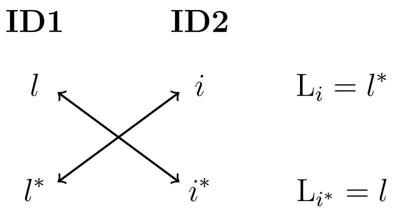

(e) After the above swapping we obtain a candidate matching vector $\mathbf{L_{cand}}$. Observe that the linking and swapping only takes place between individuals of the same sex so that mismatch does not occur. Then we update $\mathbf{L}$ using the Metropolis-Hastings acceptance ratio (ρ)

$$\rho = \frac{\pi(\mathbf{L_{cand}})\, f(\mathbf{Y}^*, \mathbf{u_{obs}} \,|\, \mathbf{L_{cand}}, \boldsymbol{\mu}_{\text{rest}})\, (b^{back}_{l^*,l} / \sum_a b^{back}_{l^*,a})}{\pi(\mathbf{L}) f(\mathbf{Y}^*, \mathbf{u_{obs}} \,|\, \mathbf{L}, \boldsymbol{\mu}_{\text{rest}})\, (b_{l,l^*} / \sum_a b_{l,a})}$$

where $\boldsymbol{\mu}_{\text{rest}}$ denotes all the remaining parameters other than $\mathbf{L}$ and $\pi(\mathbf{L})$ denotes the prior density of $\mathbf{L}$.

11. **Update ϕ (only for M_1 and M_3).** The full conditional of ϕ^* is obtained from (D.1) and is of a non-standard form

$$\pi(\phi^* \,|\, \mathbf{Y}^*, \boldsymbol{\mu}_{\text{rest}}) \propto \pi(\phi^*) \prod_{i=1}^{M} \Big(\frac{\exp(\phi^*)}{1+\exp(\phi^*)}\Big)^{z_i y_{i\cdot\cdot}} \Big(1 - \frac{\exp(\phi^*)}{1+\exp(\phi^*)}\Big)^{z_i(2n_{i\cdot} - y_{i\cdot\cdot})}$$
$$\times \prod_{j=1}^{J} \Big\{(1 - \eta_j(\mathbf{s}_i, u_i)) + \Big(1 - \frac{\exp(\phi^*)}{1+\exp(\phi^*)}\Big)^2 \eta_j(\mathbf{s}_i, u_i)\Big\}^{z_i(K - n_{ij})},$$

where $y_{i\cdot\cdot} = y^{(1)}_{i\cdot\cdot} + y^{(2*)}_{i\cdot\cdot}$ for each i and $\boldsymbol{\mu}_{\text{rest}}$ denotes all the remaining parameters other than ϕ^*. The parameter ϕ^* can be simulated using a random walk Metropolis algorithm. A normal proposal is assumed for ϕ^*. The parameters of this proposal distribution at t^{th} iteration are $\phi^{*(t-1)}$ and τ^2_ϕ, where $\phi^{*(t-1)}$ is the $(t-1)^{\text{th}}$ element in the Markov chain of ϕ^*.

12. **Update ω_0^* (only for M_1 and M_3).** The full conditional of ω_0^* is obtained from

(D.1) and is of a non-standard form

$$\pi(\omega_0^* \,|\, \mathbf{Y}^*, \boldsymbol{\mu}_{\text{rest}}) \propto \pi(\omega_0^*) \prod_{i=1}^{M} \prod_{j=1}^{J} \left(\frac{\exp(\omega_0^*)}{1+\exp(\omega_0^*)} \right)^{z_i n_{ij}} \left\{ (1-\eta_j(\mathbf{s}_i, u_i)) + \left(1 - \frac{\exp(\phi^*)}{1+\exp(\phi^*)} \right)^2 \eta_j(\mathbf{s}_i, u_i) \right\}^{z_i(K-n_{ij})},$$

where $\boldsymbol{\mu}_{\text{rest}}$ denotes all the remaining parameters other than ω_0^*. The parameter ω_0^* can be updated using a random walk Metropolis algorithm. A normal proposal is assumed for ω_0^*. The parameters of this proposal distribution at t^{th} iteration are $\omega_0^{*(t-1)}$ and τ_p^2, where $\omega_0^{*(t-1)}$ is the $(t-1)^{\text{th}}$ element in the Markov chain of ω_0^*.

13. **Update p_0^* (only for M_2 and M_4).** Under M_2, the full conditional density of p_0^* can be expressed as the following

$$\pi(p_0^* \,|\, \mathbf{Y}^*, \boldsymbol{\mu}_{\text{rest}}) \propto \pi^*(p_0^*) \prod_{i=1}^{M} \prod_{j=1}^{J} \left(\frac{\exp(p_0^*)}{1+\exp(p_0^*)} \right)^{z_i y_{ij\cdot}} (1 - p_j(\mathbf{s}_i, u_i))^{z_i(2K - y_{ij\cdot})}.$$

where $\boldsymbol{\mu}_{\text{rest}}$ denotes all the remaining parameters other than p_0^*. The full conditional of p_0^* is of a non-standard form and can be updated using a random walk Metropolis algorithm. A normal proposal is assumed for p_0^*. The parameters of this proposal distribution at t^{th} iteration are $p_0^{*(t-1)}$ and τ_λ^2, where $p_0^{*(t-1)}$ is the $(t-1)^{\text{th}}$ element in the Markov chain of p_0^*. The parameter p_0^* can be updated similarly for M_4.

14. Repeat the above steps (1) - (13) until convergence of each parameter.

E Results of the simulation study

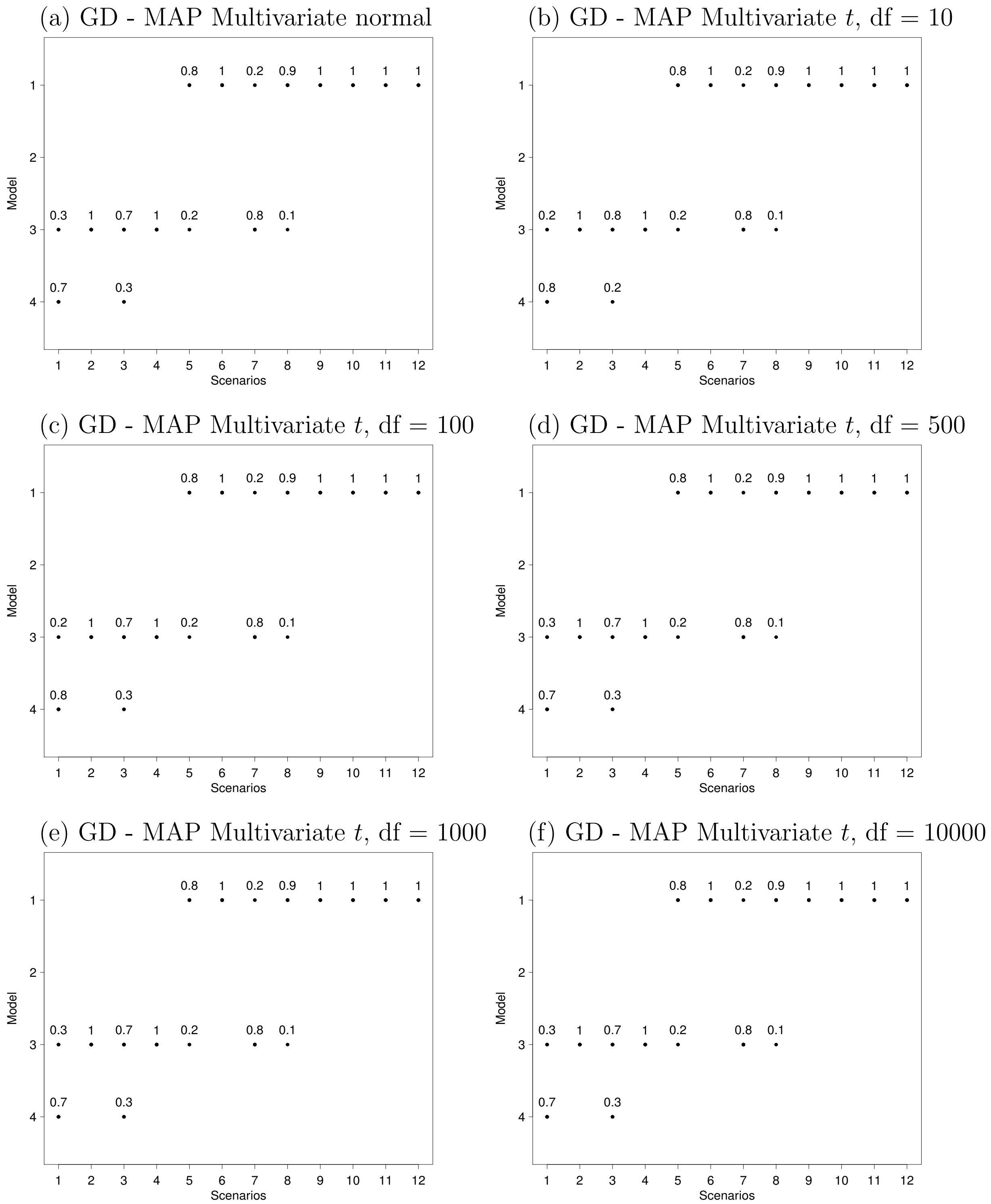

Figure 1: The proportion of times Bayes factor favours any particular model using the MAP approximation approach. Plot (a): Gelfand-Dey estimator with a multivariate normal density for g. Plots (b)-(f) : Gelfand-Dey estimator with five different choices for g, viz., densities of multivariate-t distribution with degrees of freedom 10, 100, 500, 1000, 10000 respectively.

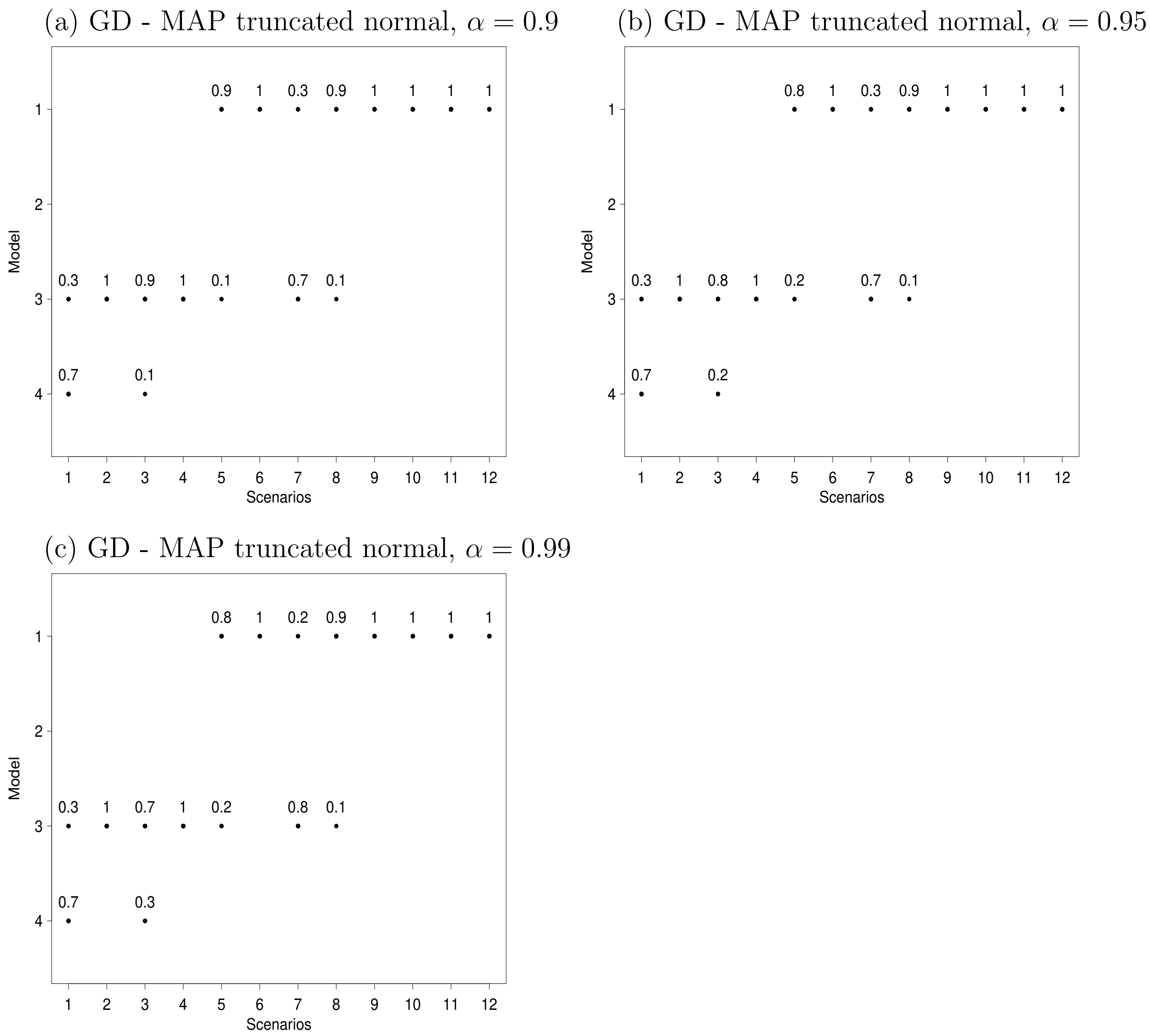

Figure 2: The proportion of times Bayes factor favours any particular model using the MAP approximation approach. Plots (a) - (c) are obtained by computing Gelfand-Dey estimator with three different choices for g, viz., truncated normal density with $\alpha = 0.9, 0.95, 0.99$.

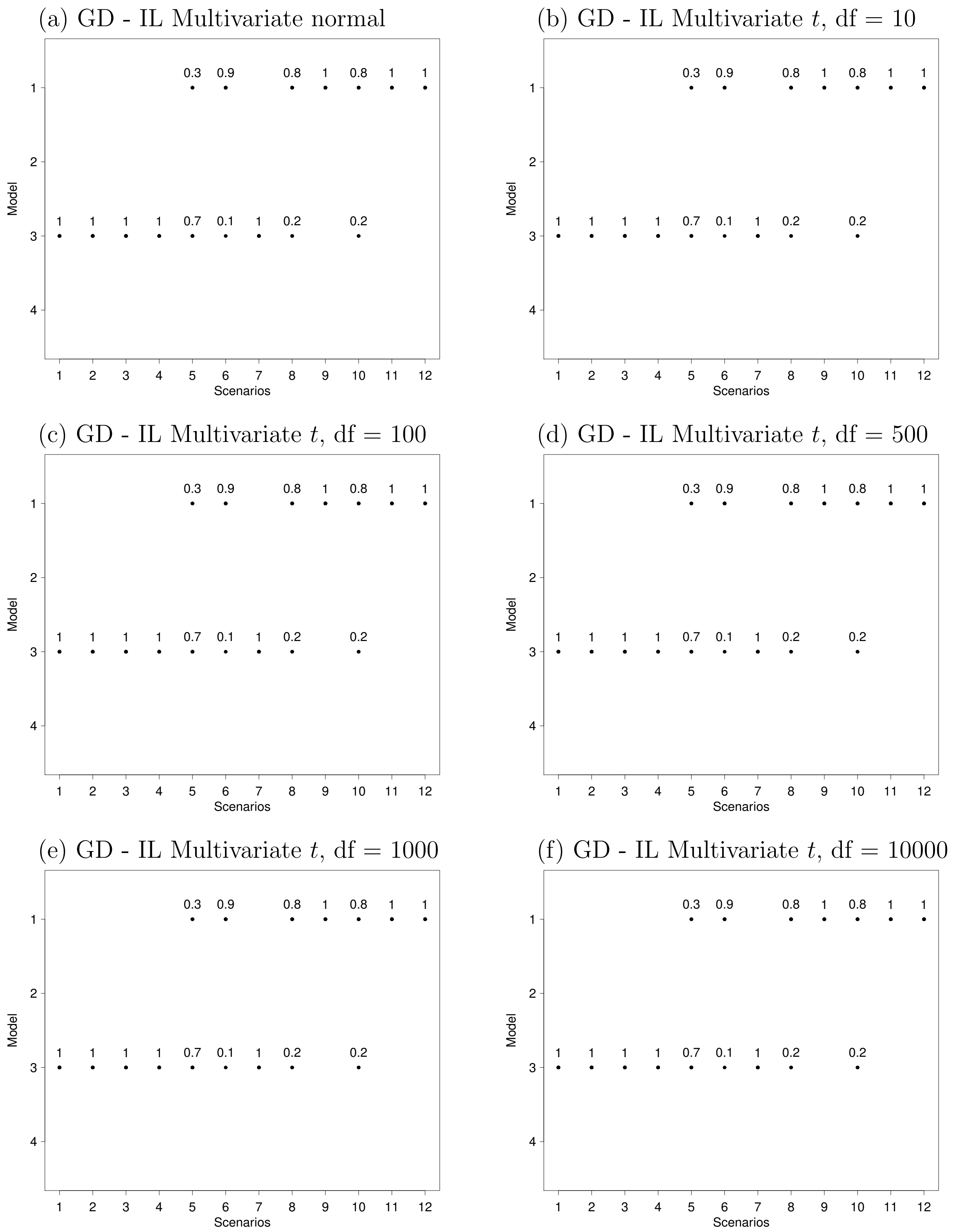

Figure 3: The proportion of times Bayes factor favours any particular model using the integrated likelihood approximation (IL) approach. Plot (a): Gelfand-Dey estimator with a multivariate normal density for g. Plots (b)-(f) : Gelfand-Dey estimator with five different choices for g, viz., densities of multivariate-t distribution with degrees of freedom 10, 100, 500, 1000, 10000 respectively.

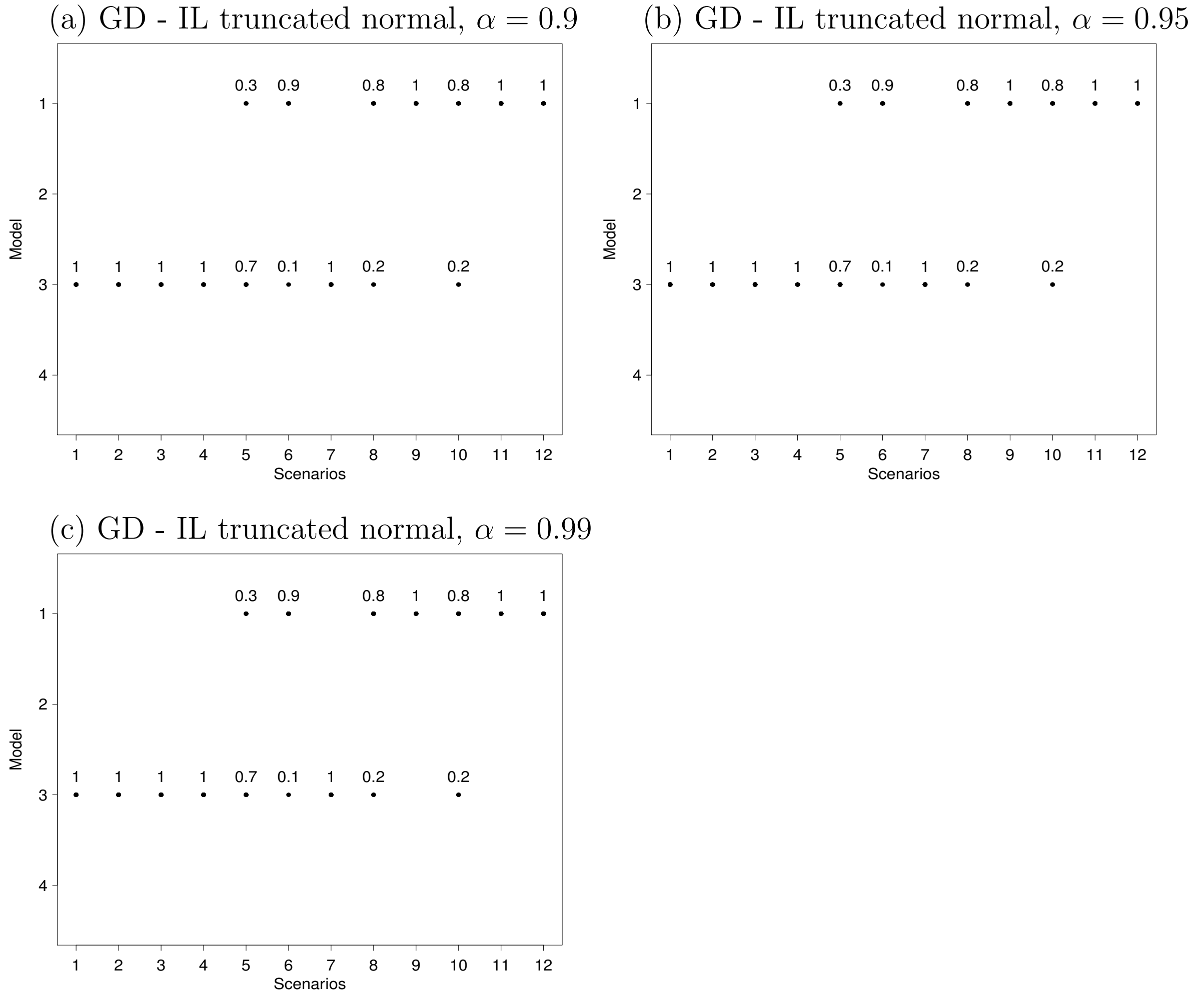

Figure 4: The proportion of times Bayes factor favours any particular model using the integrated likelihood approximation approach. Plots (a) - (c) : Gelfand-Dey estimator with three different choices for g, viz., truncated normal density with $\alpha = 0.9, 0.95, 0.99$.

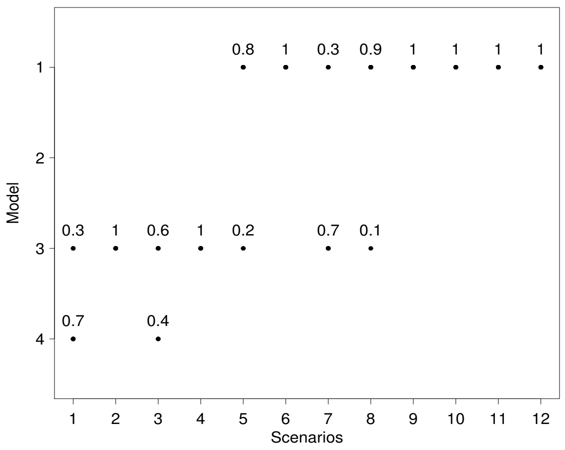

Figure 5: The proportion of times Bayes factor (using the harmonic mean estimator) favours any particular model.

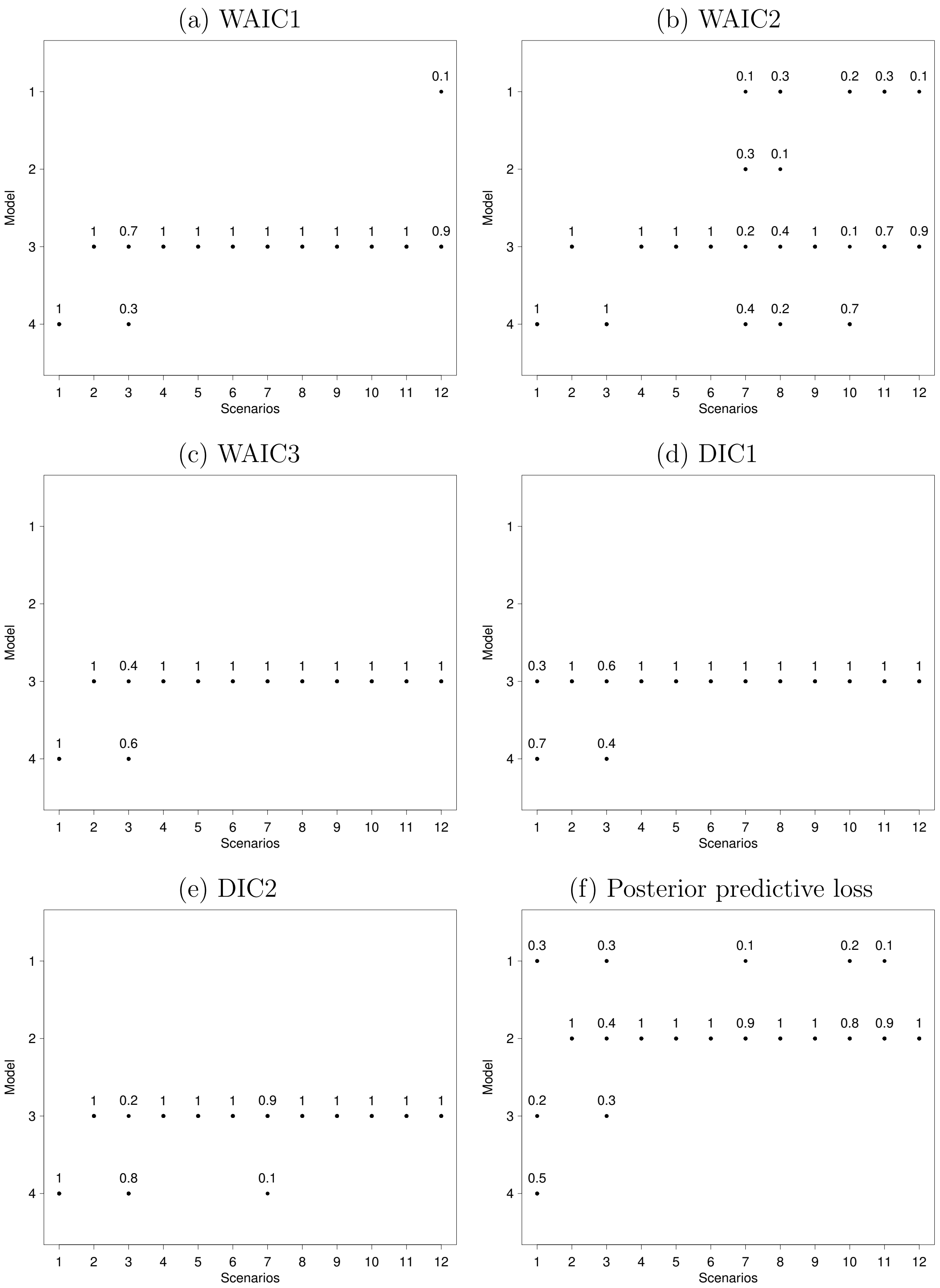

Figure 6: The proportion of time a model selection method favours any particular model. Plots (a)-(f) correspond to WAIC1, WAIC2, WAIC3, DIC1, DIC2 and posterior predictive loss respectively.

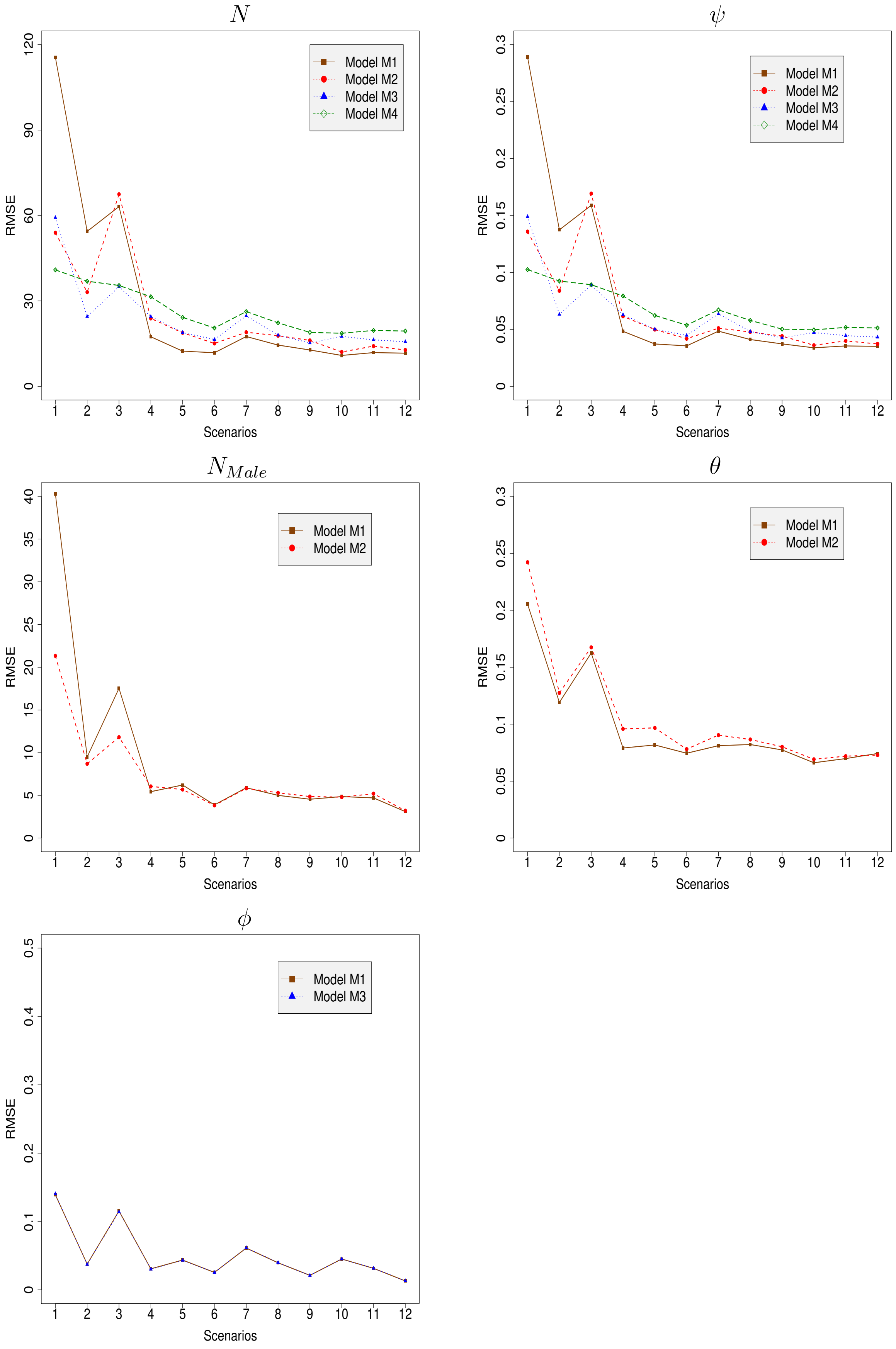

Figure 7: Plot of average RMSE estimates of N, ψ, N_{Male}, θ and ϕ over different simulation scenarios.

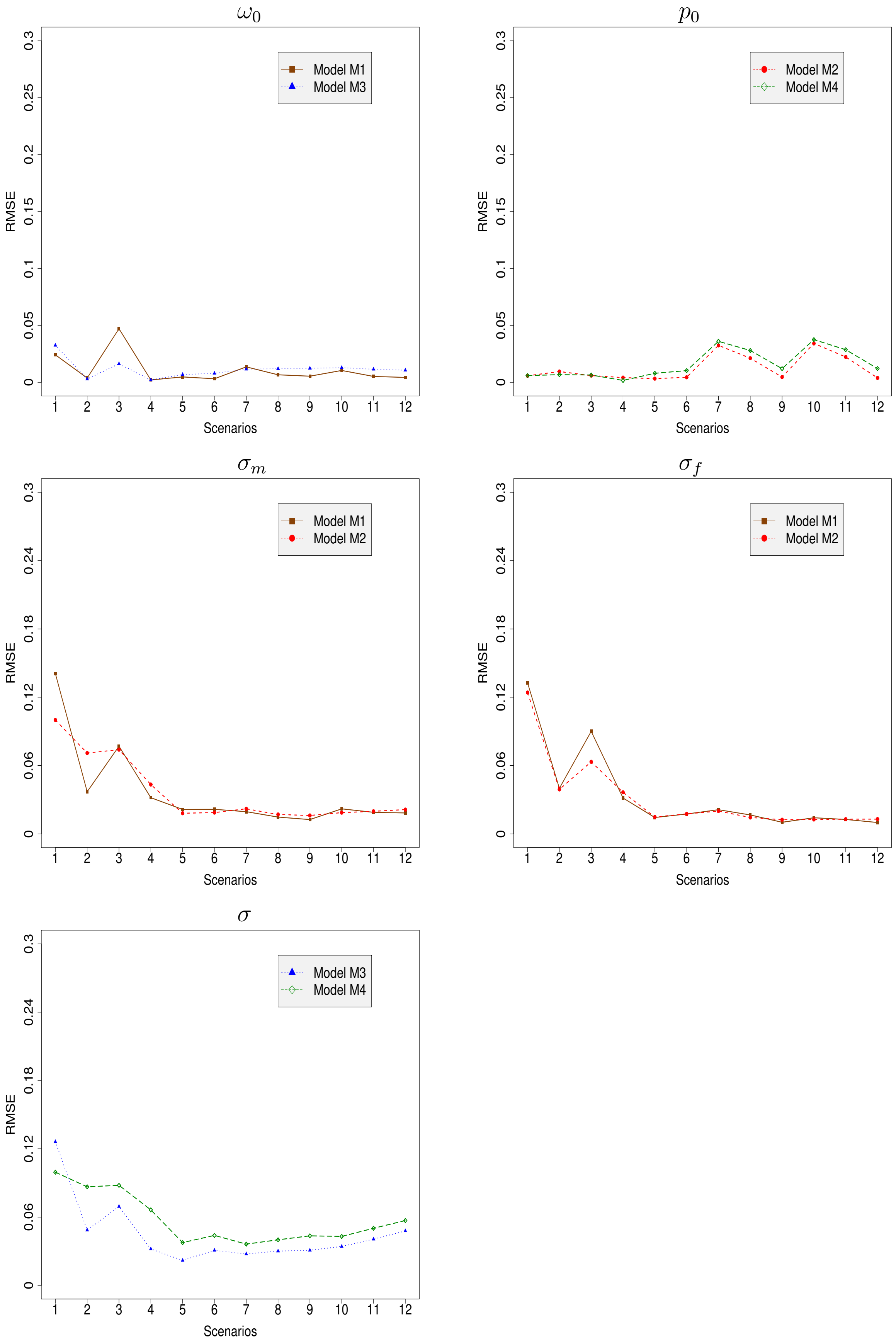

Figure 8: Plot of average RMSE estimates of ω_0, p_0, σ_m, σ_f and σ over different simulation scenarios.

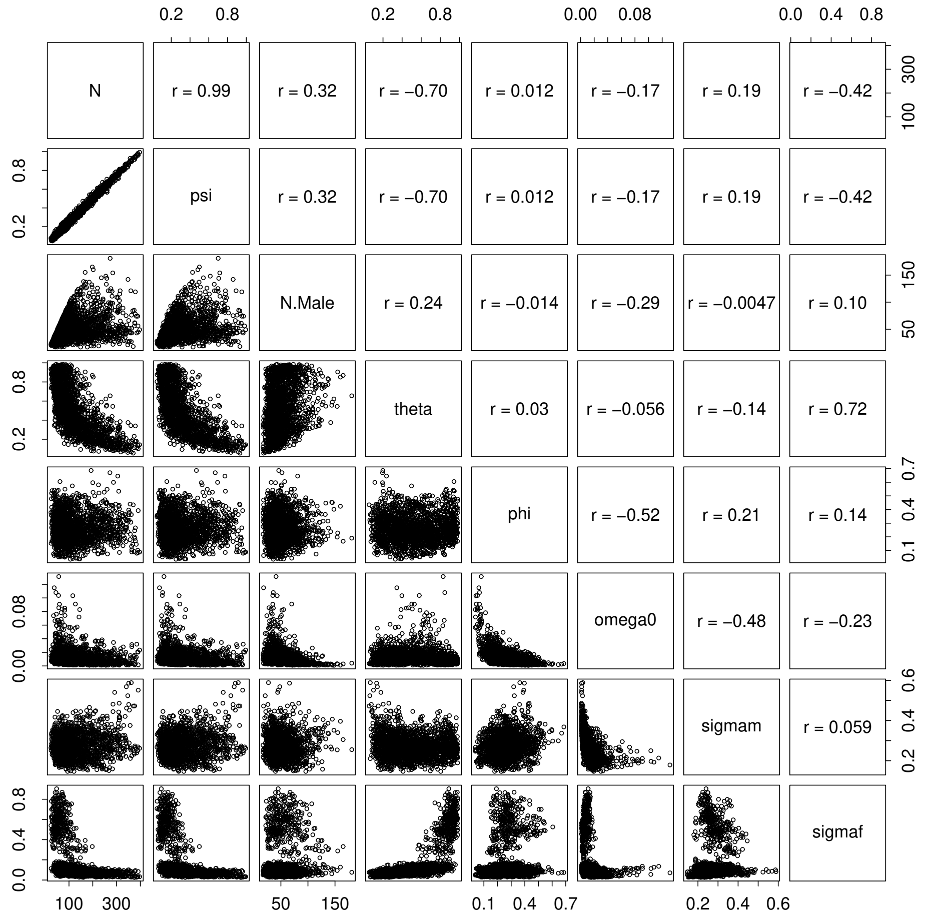

Figure 9: Scatter plot of the parameters obtained from MCMC chains of a simulated data analysis of M_1 corresponding to scenario 1. Here r denotes the correlation coefficient and is computed by using the MCMC chains of the respective parameters.

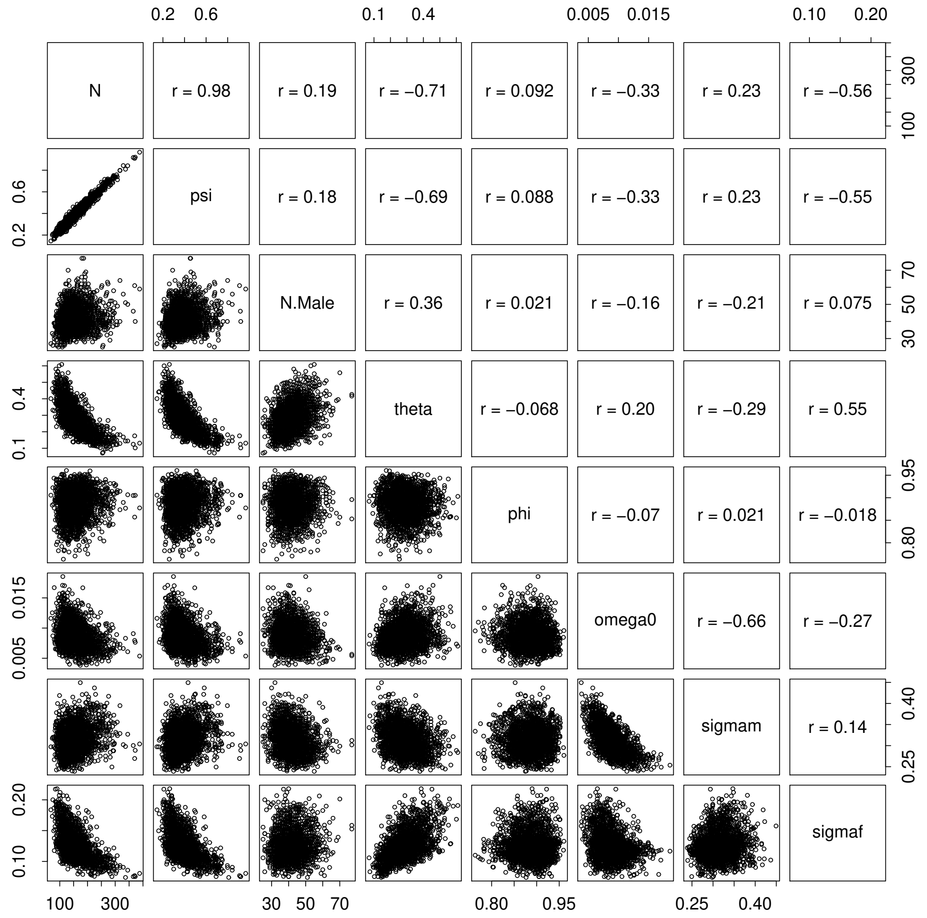

Figure 10: Scatter plot of the parameters obtained from MCMC chains of a simulated data analysis of M_1 corresponding to scenario 2. Here r denotes the correlation coefficient and is computed by using the MCMC chains of the respective parameters.

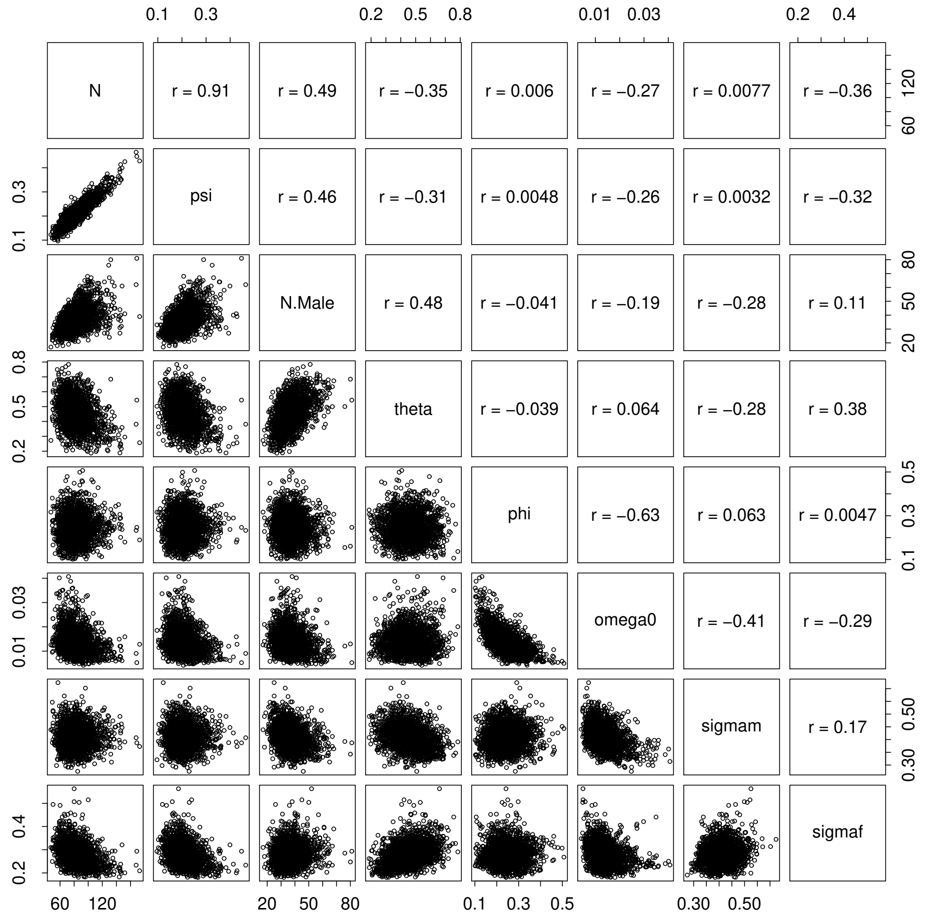

Figure 11: Scatter plot of the parameters obtained from MCMC chains of a simulated data analysis of M_1 corresponding to scenario 3. Here r denotes the correlation coefficient and is computed by using the MCMC chains of the respective parameters.

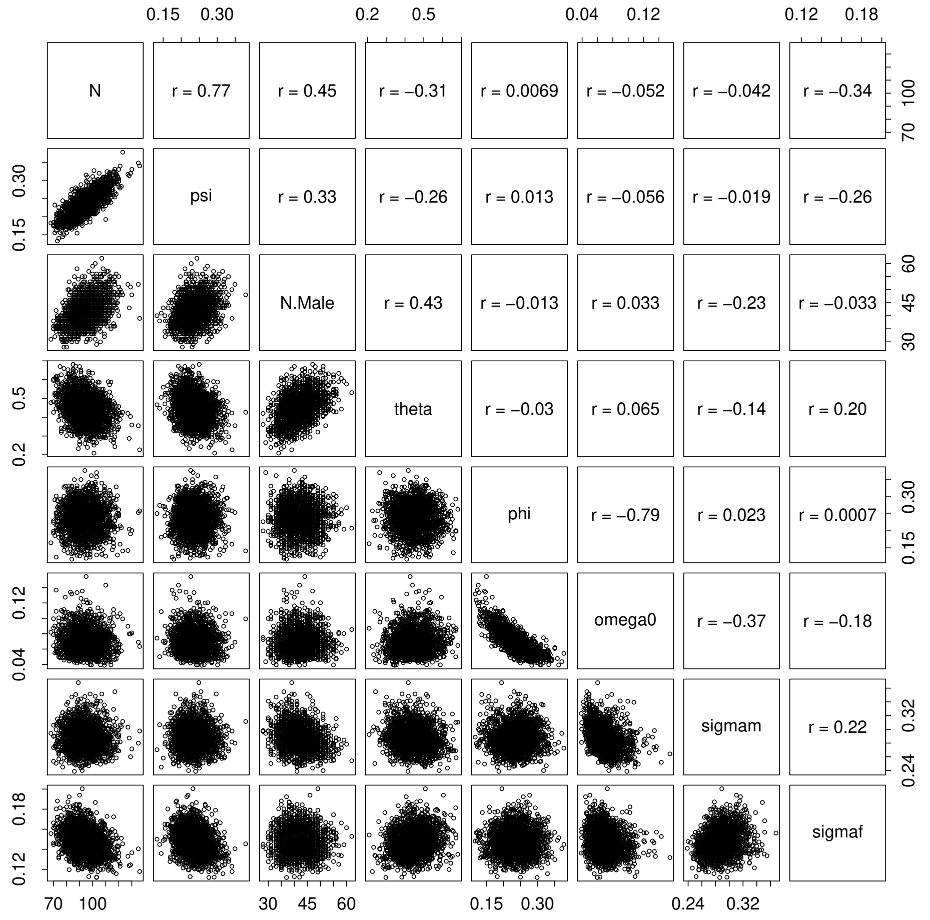

Figure 12: Scatter plot of the parameters obtained from MCMC chains of a simulated data analysis of M_1 corresponding to scenario 7. Here r denotes the correlation coefficient and is computed by using the MCMC chains of the respective parameters.

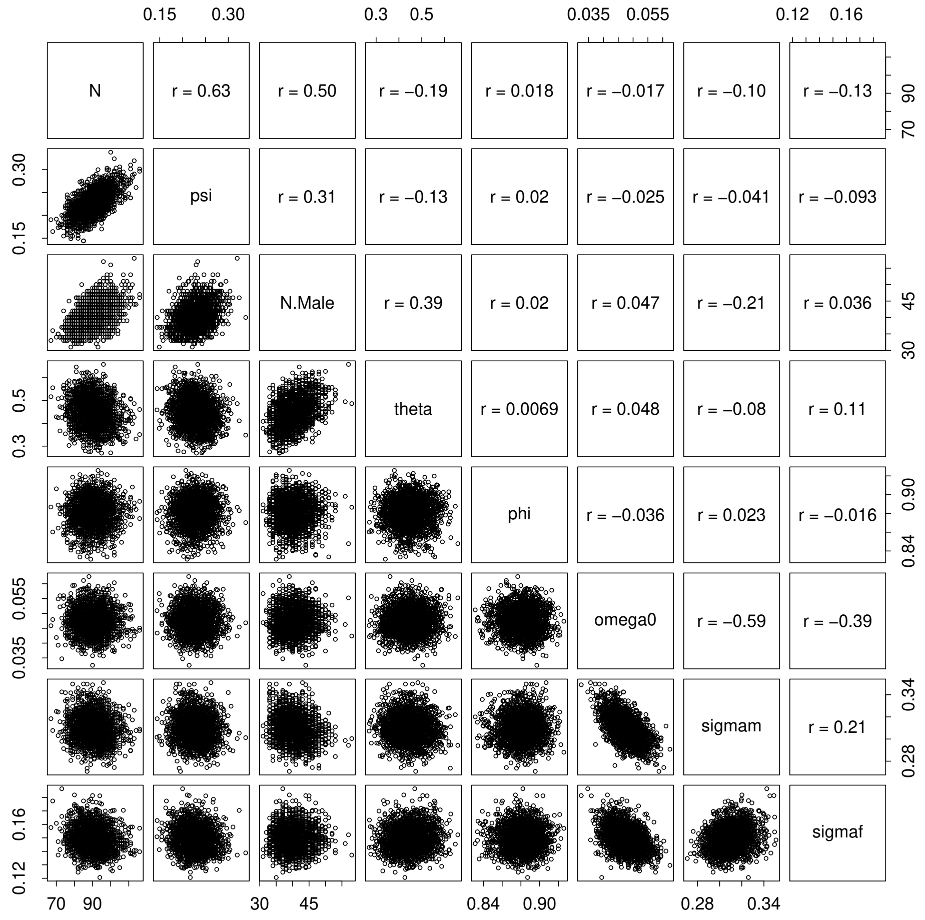

Figure 13: Scatter plot of the parameters obtained from MCMC chains of a simulated data analysis of M_1 corresponding to scenario 9. Here r denotes the correlation coefficient and is computed by using the MCMC chains of the respective parameters.

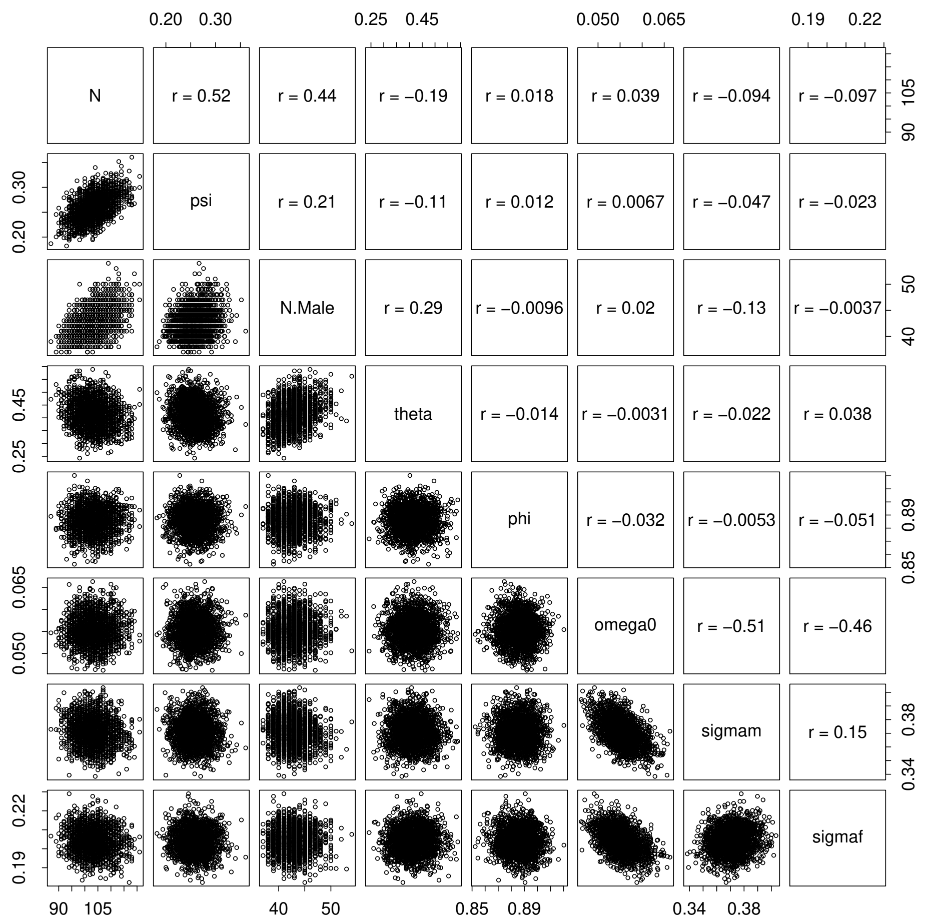

Figure 14: Scatter plot of the parameters obtained from MCMC chains of a simulated data analysis of M_1 corresponding to scenario 12. Here r denotes the correlation coefficient and is computed by using the MCMC chains of the respective parameters.

References

Geweke, J. (1999). Using simulation methods for Bayesian econometric models: inference, development, and communication. *Econometric reviews*, 18(1):1–73.
